# Supplementary material for: Sequencing of Historical Isolates, K-mer Mining and High Serological Cross-Reactivity with Ross River Virus Argue against the Presence of Getah Virus in Australia
Source: Pathogens. 2020 Oct 16;9(10):848. doi: 10.3390/pathogens9100848 (PMC7650646; doi:10.3390/pathogens9100848)
Supplement: Supplementary file 1 [file pathogens-09-00848-s001.zip › pathogens-948433-supplementary.docx]

**
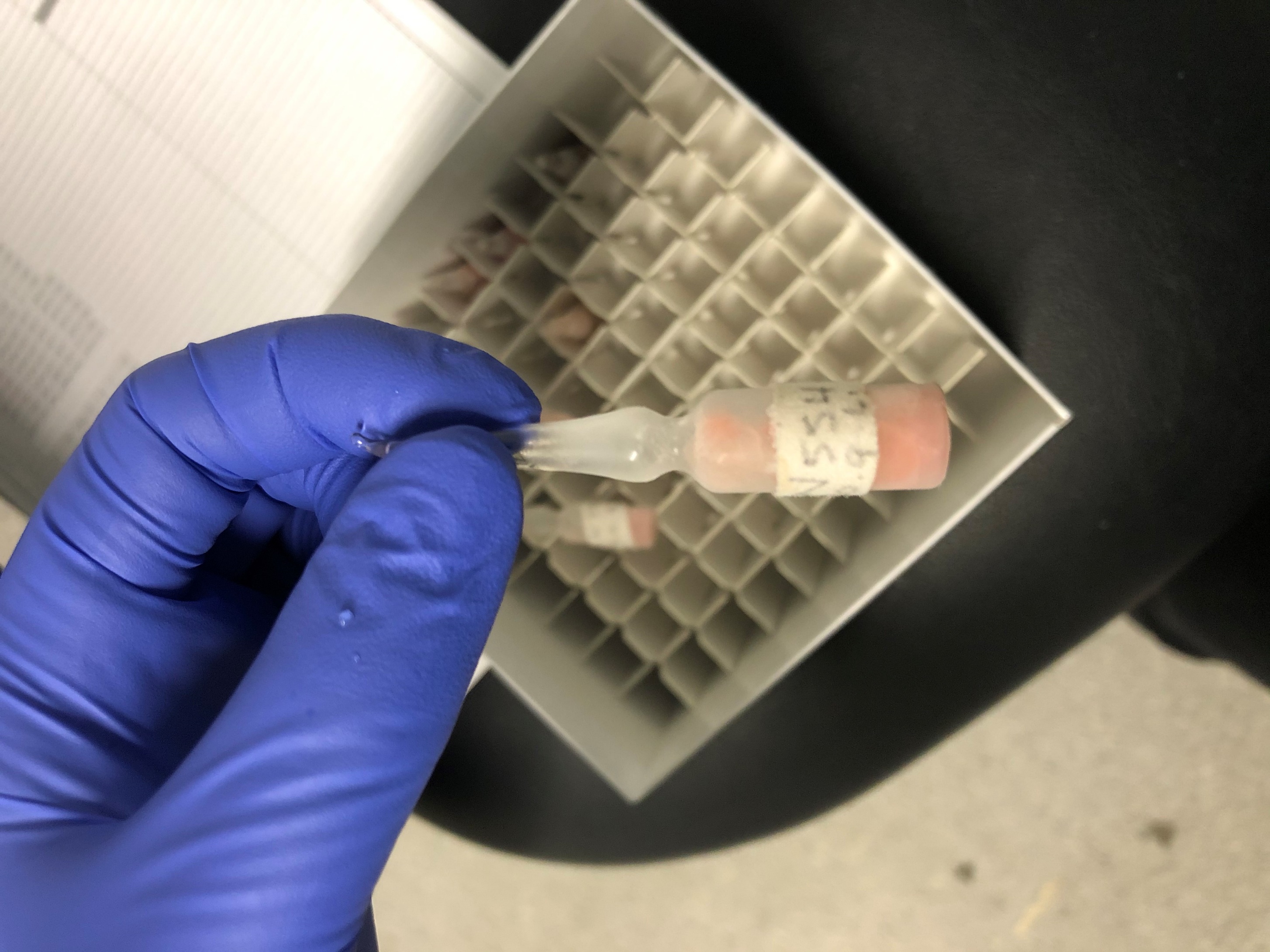

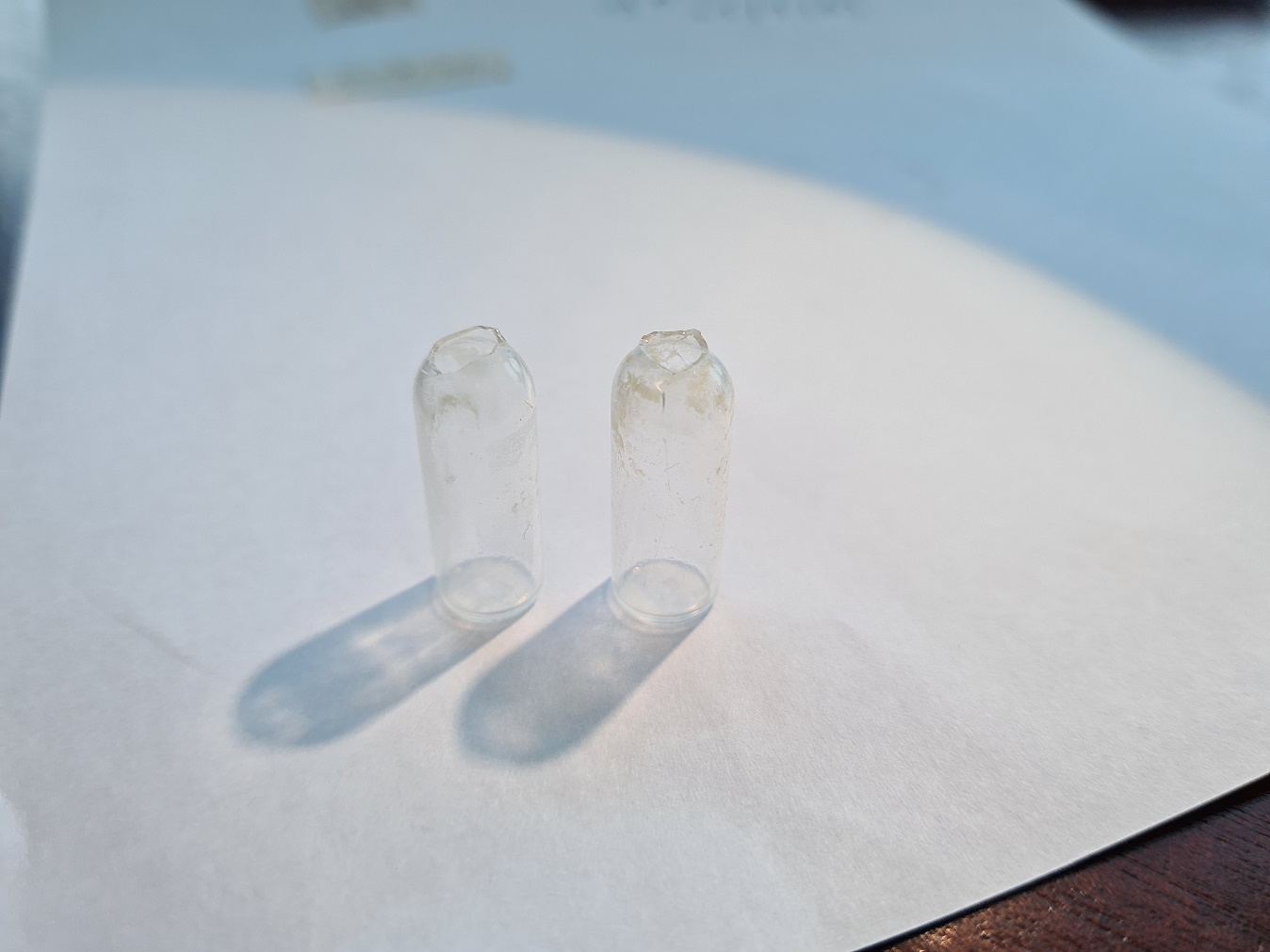

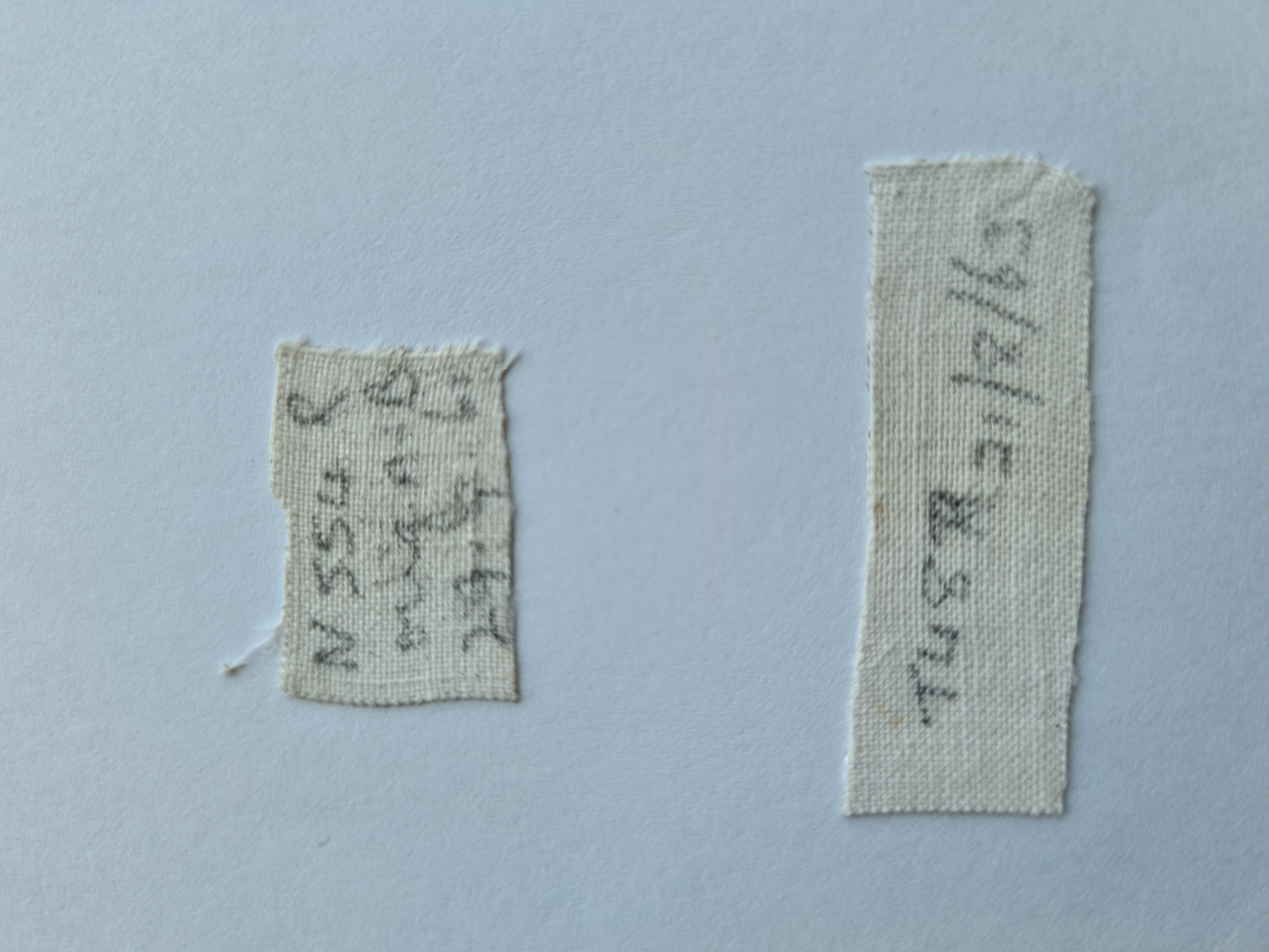
**

**Figure S1**. Photographs of the glass vials of N554 frozen in 1961. The vials were kept as part of the “Doherty Virus Collection” created by Prof. Ralph Doherty, the first director of the Queensland Institute of Medical Research.

**Query N554**. Length: 11648

Sbjct Getah virus strain MM2021, complete genome

Sequence ID: MN849355.1 Length: 11690

Range 1: 1 to 11648

Score:21461 bits(11621), Expect:0.0,

Identities:11639/11648(99%), Gaps:0/11648(0%), Strand: Plus/Plus

Query 1 ATGGCGGACGTGTGACATCACCGTTCGCTCTTTCTAGGATCCTTTGCTACTCCACATAGT 60

Sbjct 1 ............................................................ 60

Query 61 GAGAGACAGACAACCCAAATGAAGGTAACCGTGGACGTTGAGGCTGACAGCCCATTCCTT 120

Sbjct 61 ............................................................ 120

Query 121 AAGGCCCTTCAGAAGGCGTTTCCCGCCTTTGAGGTTGAATCACAGCAGGTCACACCGAAT 180

Sbjct 121 ............................................................ 180

Query 181 GACCATGCTAACGCCAGAGCATTTTCGCATCTGGCTACCAAACTGATTGAGCAAGAGGTT 240

Sbjct 181 ............................................................ 240

Query 241 CCAACAGGCGTCACCATCCTGGATGTGGGTAGTGCACCCGCAAGGAGGTTGATGTCTGAC 300

Sbjct 241 ............................................................ 300

Query 301 CACACCTACCACTGCATCTGCCCTATGAAAAGTGCGGAAGACCCAGAGAGGTTGGCGAAT 360

Sbjct 301 ............................................................ 360

Query 361 TACGCTCGAAAGCTGGCGAAAGCATCGGGGACTGTGCTAGACAAGAATGTGTCCGGGAAG 420

Sbjct 361 ............................................................ 420

Query 421 ATAACGGACCTTCAAGACGTCATGGCCACTCCAGACTTGGAATCCCCGACTTTTTGCCTG 480

Sbjct 421 ............................................................ 480

Query 481 CACACCGACGAGACGTGCCGCACTAGGGCTGAGGTCGCTGTGTACCAGGACGTATACGCT 540

Sbjct 481 ............................................................ 540

Query 541 GTGCACGCACCGACGTCTCTGTACCACCAGGCAATTAAAGGTGTAAGGACGGCGTATTGG 600

Sbjct 541 ............................................................ 600

Query 601 ATTGGATTCGACACCACTCCATTCATGTTCGAGGCACTAGCGGGCGCGTACCCTGCATAC 660

Sbjct 601 ............................................................ 660

Query 661 TCGACCAATTGGGCAGATGAGCAAGTGCTGCAGGCTCGTAACATCGGCCTGTGCGCGACA 720

Sbjct 661 ............................................................ 720

Query 721 GGCCTCTCTGAGGGGCGTCGCGGCAAACTCTCCATCATGAGAAAGAAGTGCTTGCGACCG 780

Sbjct 721 ............................................................ 780

Query 781 AGCGACAGAGTAATGTTTTCGGTCGGGTCCACCTTGTACACCGAGAGCCGAAAGCTGCTG 840

Sbjct 781 ............................................................ 840

Query 841 CGCAGCTGGCATTTACCTTCCGTGTTTCACCTGAAGGGTAAGAACAGTTTTACCTGCAGG 900

Sbjct 841 ............................................................ 900

Query 901 TGTGACACGGTGGTGTCATGCGAAGGTTATGTGGTAAAGAAGATCACCATAAGCCCGGGC 960

Sbjct 901 ............................................................ 960

Query 961 ATATATGGAAAAACAGTTGACTACGCAGTTACCCATCACGCAGAGGGTTTCCTGATGTGC 1020

Sbjct 961 ............................................................ 1020

Query 1021 AAGATCACTGATACAGTCAGAGGAGAAAGAGTCTCTTTCCCGGTCTGTACCTATGTGCCT 1080

Sbjct 1021 ............................................................ 1080

Query 1081 GCAACCATATGCGACCAGATGACGGGTATACTCGCCACCGACGTGACACCGGAGGATGCC 1140

Sbjct 1081 ............................................................ 1140

Query 1141 CAGAAGCTCCTGGTCGGATTGAACCAACGCATAGTGGTGAATGGTAGGACGCAAAGAAAC 1200

Sbjct 1141 ............................................................ 1200

Query 1201 ACAAATACAATGAAAAACTACCTACTGCCAGTGGTAGCGCAAGCATTCAGTAAATGGGCG 1260

Sbjct 1201 ............................................................ 1260

Query 1261 CGTGAGGCGCGCGCAGACATGGAGGATGAAAAACCCCTAGGCACCAGAGAACGCACGTTG 1320

Sbjct 1261 ............................................................ 1320

Query 1321 ACGTGTTGTTGCCTGTGGGCGTTTAAAAGCCACAAAATCCACACCATGTATAAGCGGCCT 1380

Sbjct 1321 ............................................................ 1380

Query 1381 GAAACGCAGACTATCGTCAAAGTGCCTTCTACTTTTGACTCCTTTGTGATACCGAGCCTG 1440

Sbjct 1381 ............................................................ 1440

Query 1441 TGGTCATCCAGCCTTTCCATGGGTATCAGACAGAGGATCGAACTGCTTCTCAGCGCAAGA 1500

Sbjct 1441 ............................................................ 1500

Query 1501 ATGGCCCAAAGCCTACCATACTCAGGAGACCGCACTGAAGCTCGAGCGGCTGAAGAAGAA 1560

Sbjct 1501 ............................................................ 1560

Query 1561 GAGAAAGAGGCGCAGGAGGCAGAACTTACGAGGGCAGCGCTGCCACCACTAGTGAGTGGC 1620

Sbjct 1561 ............................................................ 1620

Query 1621 TCTTGCGCTGACGATATCACCCAGGTAGATGTAGAGGAATTAACCTTCAGAGCCGGAGCC 1680

Sbjct 1621 ............................................................ 1680

Query 1681 GGGGTTGTGGAAACACCCAGGAACGCCCTGAAGGTCACACCGCAAGCACACGACCACCTC 1740

Sbjct 1681 ............................................................ 1740

Query 1741 ATAGGCTCCTACTTGATCCTTTCCCCCCAAACGGTCTTGAAAAGTGAGAAGCTGGCACCC 1800

Sbjct 1741 ............................................................ 1800

Query 1801 ATCCATCCTCTTGCCGAGCAAGTCACGGTCATGACCCACTCTGGGAGATCCGGCAGATAC 1860

Sbjct 1801 ............................................................ 1860

Query 1861 CCAGTCGACAAGTACGACGGACGGGTATTGATCCCAACAGGAGCGGCCATCCCAGTGAGT 1920

Sbjct 1861 ............................................................ 1920

Query 1921 GAGTTCCAGGCACTCAGCGAGAGCGCAACCATGGTGTACAATGAGAGGGAATTTATAAAT 1980

Sbjct 1921 ............................................................ 1980

Query 1981 CGTAAACTACACCACATAGCGCTATACGGGCCAGCCTTGAACACCGACGAGGAAAGCTAC 2040

Sbjct 1981 ............................................................ 2040

Query 2041 GAAAAAGTGAGAGCTGAGAGGGCAGAGACAGAGTATGTGTTCGACGTGGACAAGAAGGCA 2100

Sbjct 2041 ............................................................ 2100

Query 2101 TGCATCAAGAAGGAGGAGGCATCAGGCCTTGTGTTAACAGGGGACCTAATCAATCCACCT 2160

Sbjct 2101 ............................................................ 2160

Query 2161 TTCCACGAATTCGCATACGAAGGACTCAAGATCCGCCCAGCAGCCCCGTACCACACGACG 2220

Sbjct 2161 ............................................................ 2220

Query 2221 ATCATTGGGGTGTTTGGCGTTCCAGGTTCGGGCAAGTCGGCTATCATTAAGAACATGGTG 2280

Sbjct 2221 ............................................................ 2280

Query 2281 ACGACCCGCGATCTAGTGGCCAGTGGAAAGAAGGAGAACTGCCAAGAGATCATGAACGAC 2340

Sbjct 2281 ............................................................ 2340

Query 2341 GTAAAGAGGCAACGCGGGTTGGATGTGACCGCTAGAACCGTCGACTCAATCTTATTAAAT 2400

Sbjct 2341 ............................................................ 2400

Query 2401 GGGTGCAAGAGAGGCGTAGAAAACCTTTACGTCGATGAGGCGTTCGCGTGCCACTCGGGT 2460

Sbjct 2401 ............................................................ 2460

Query 2461 ACTTTGCTAGCGCTTATTGCGCTAGTGAGACCGTCAGGTAAGGTAGTACTGTGCGGCGAC 2520

Sbjct 2461 ............................................................ 2520

Query 2521 CCTAAGCAGTGTGGCTTCTTCAACTTGATGCAACTGAAGGTGCACTACAACCACAACATC 2580

Sbjct 2521 ............................................................ 2580

Query 2581 TGTACAAGGGTGCTCCATAAGAGCATCTCCAGAAGATGCACACTACCTGTCACGGCGATC 2640

Sbjct 2581 ............................................................ 2640

Query 2641 GTGTCCACCTTGCACTACCAAGGGAAGATGAGAACGACGAACCGATGCAATACTCCTATT 2700

Sbjct 2641 ............................................................ 2700

Query 2701 CAGATCGACACCACCGGTTCCTCCAAACCGGCCTCAGGAGATATCGTGTTAACGTGTTTC 2760

Sbjct 2701 ............................................................ 2760

Query 2761 CGCGGCTGGGTGAAACAACTGCAAATCGACTATCGTGGACACGAGGTGATGACCGCAGCT 2820

Sbjct 2761 ............................................................ 2820

Query 2821 GCTTCTCAGGGTCTGACAAGGAAAGGCGTGTACGCCGTGAGACAGAAAGTGAACGAAAAC 2880

Sbjct 2821 ............................................................ 2880

Query 2881 CCACTGTACTCACCTCTGTCAGAGCACGTCAATGTGCTGTTGACCCGAACTGAAAACCGA 2940

Sbjct 2881 ............................................................ 2940

Query 2941 CTGGTGTGGAAAACACTGTCGGGTGACCCGTGGATAAAGGTGTTAACCAATGTTCCACGA 3000

Sbjct 2941 ............................................................ 3000

Query 3001 GGAGATTTCAGTGCGACTTTGGAGGAATGGCAAGAAGAACATGAAGGTATCATGAGAGTG 3060

Sbjct 3001 ............................................................ 3060

Query 3061 TTGAACGAGCGACCAGCGGAGGTTGATCCATTCCAAAATAAGGCCAAAGTGTGCTGGGCA 3120

Sbjct 3061 ............................................................ 3120

Query 3121 AAATGTCTGGTGCAAGTTCTTGAGACAGCCGGAATACGTATGACGGCAGATGAATGGAAC 3180

Sbjct 3121 ............................................................ 3180

Query 3181 ACCATCTTGGCTTTTAGAGAAGACAGAGCATACTCACCAGAAGTCGCTCTCAATGAGATC 3240

Sbjct 3181 ............................................................ 3240

Query 3241 TGCACTCGTTACTACGGCGTCGACCTAGACAGCGGCCTATTCTCAGCGCAGTCAGTCTCC 3300

Sbjct 3241 ............................................................ 3300

Query 3301 CTCTTTTATGAGAACAACCACTGGGACAACAGGCCTGGAGGACGTATGTATGGGTTCAAC 3360

Sbjct 3301 ............................................................ 3360

Query 3361 CACGAAGTAGCCAGGAAATATGCAGCTAGGTTTCCATTTCTACGTGGCAACATGAACTCG 3420

Sbjct 3361 ............................................................ 3420

Query 3421 GGGCTACAACTAAACGTCCCTGAGAGGAAGCTCCAACCTTTTAGCGCTGAATGCAACATA 3480

Sbjct 3421 ............................................................ 3480

Query 3481 GTACCATCCAATCGCCGGTTGCCGCATGCTCTAGTCACCACTTATCAGCAGTGCCGCGGG 3540

Sbjct 3481 ............................................................ 3540

Query 3541 GAGAGGGTAGAGTGGTTGCTGAAAAAGATCCCTGGCCACCAAATGTTACTTGTAAGCGAG 3600

Sbjct 3541 ............................................................ 3600

Query 3601 TACAACCTGGTGATACCTCACAAAAGAGTCTTCTGGATTGCACCTCCGAGGGTGTCAGGC 3660

Sbjct 3601 ............................................................ 3660

Query 3661 GCGGACCGCACGTACGACTTGGACCTAGGGTTACCTATGGATGCAGGTCGTTACGATTTG 3720

Sbjct 3661 ............................................................ 3720

Query 3721 GTATTCGTCAACATCCATACCGAGTACCGGCAGCACCACTACCAACAATGCGTCGACCAT 3780

Sbjct 3721 ............................................................ 3780

Query 3781 TCAATGCGCCTGCAGATGCTGGGAGGGGATTCACTACACCTGCTTAGACCAGGAGGCTCG 3840

Sbjct 3781 ............................................................ 3840

Query 3841 CTGCTGATGAGAGCATATGGTTATGCAGACAGAGTCAGCGAGATGGTGGTGACAGCCCTG 3900

Sbjct 3841 ............................................................ 3900

Query 3901 GCTAGGAAATTCTCGGCGTTCCGTGTCCTGAGGCCAGCGTGTGTGACGAGCAACACAGAA 3960

Sbjct 3901 ............................................................ 3960

Query 3961 GTGTTCCTGCTGTTTTCTAACTTCGATAACGGCAGGAGAGCGGTAACCTTGCACCAAGCT 4020

Sbjct 3961 ............................................................ 4020

Query 4021 AATCAGAGACTTAGCTCGATGTATGCCTGCAACGGACTGCACACTGCAGGCTGTGCACCG 4080

Sbjct 4021 ............................................................ 4080

Query 4081 TCATACAGGGTTCGCCGCGCTGATATATCAGGACACGGTGAGGAAGCGGTCGTAAATGCT 4140

Sbjct 4081 ............................................................ 4140

Query 4141 GCCAACGCCAAAGGTACCGTGAGCGATGGAGTGTGTAGGGCGGTCGCTAAGAAGTGGCCA 4200

Sbjct 4141 ............................................................ 4200

Query 4201 TCATCATTCAAAGGGGCTGCAACTCCAGTCGGCACAGCCAAGATGATCCGCGCTGATGGC 4260

Sbjct 4201 ............................................................ 4260

Query 4261 ATGACCGTAATCCACGCAGTGGGACCGAATTTCTCCACCGTAACAGAAGCTGAAGGGGAC 4320

Sbjct 4261 ............................................................ 4320

Query 4321 AGAGAGCTAGCGGCCGCGTACCGAGCTGTGGCTAGCATAATTAGTACTAACAACATAAAG 4380

Sbjct 4321 ............................................................ 4380

Query 4381 AGCGTCGCAGTACCGCTGCTGTCCACAGGCACCTTCTCCGGCGGTAAGGACAGAGTGATG 4440

Sbjct 4381 ............................................................ 4440

Query 4441 CAGTCCTTGAACCACTTATTCACGGCGTTGGATGCCACCGATGCAGACGTGGTTATCTAC 4500

Sbjct 4441 ............................................................ 4500

Query 4501 TGCAGAGACAAAAACTGGGAAAAGAAGATTCAGGAAGCCATCGACAGGCGGACGGCAATA 4560

Sbjct 4501 ............................................................ 4560

Query 4561 GAGCTCGTATCTGAAGACGTGACTTTGGAAACCGACCTGGTTAGGGTACACCCGGATAGT 4620

Sbjct 4561 ...................................T........................ 4620

Query 4621 TGCTTAGTCGGCAGAAACGGTTACAGCGCAACTGACGGTAAGCTATACTCTTACCTTGAG 4680

Sbjct 4621 ............................................................ 4680

Query 4681 GGCACGAGGTTTCACCAGACGGCGGTCGACATGGCTGAAATATCAACTTTATGGCCACGA 4740

Sbjct 4681 ............................................................ 4740

Query 4741 CTCCAAGACGCTAACGAGCAGATCTGCCTGTACGCCCTAGGGGAGACGATGGACAGCATA 4800

Sbjct 4741 ............................................................ 4800

Query 4801 CGCACCAAATGCCCAGTAGAAGACGCCGATTCGTCTACGCCGCCGAAAACGGTACCATGT 4860

Sbjct 4801 ............................................................ 4860

Query 4861 TTATGTCGGTATGCGATGACCGCAGAGCGAGTTGCCAGACTTAGGATGAACAACACCAAA 4920

Sbjct 4861 ............................................................ 4920

Query 4921 AACATCATCGTGTGCTCCTCCTTTCCATTGCCGAAGTACAGGATAGAAGGCGTGCAGAAG 4980

Sbjct 4921 ............................................................ 4980

Query 4981 GTGAAGTGTGACCGAGTGCTAATTTTTGATCAGACCGTCCCGTCACTAGTAAGTCCCAGA 5040

Sbjct 4981 ............................................................ 5040

Query 5041 AAGTACATACAGCAGCTGCCGGAACAGCTGGATAATGTGAGCCTGACTTCTGCGACGTCG 5100

Sbjct 5041 ............................................................ 5100

Query 5101 ACGGGATCCGCTTGGTCGTTCCCATCGGAAACGACCTACGAAACTATGGAAGTCGTAGCC 5160

Sbjct 5101 ............................................................ 5160

Query 5161 GAGGTACACACCGAACCTCCGATCCCTCCGCCTCGTAGACGTAGAGCAGCCGTCGTCCAA 5220

Sbjct 5161 ............................................................ 5220

Query 5221 CTTAGACAAGACCTGGAAGTCACCGAGGAGATCGAACCGTACGCGATACAGCAAGCAGAG 5280

Sbjct 5221 ............................................................ 5280

Query 5281 ATCATGGTCATGGAGAGGGTCGCGACGACAGATATACGCGCTATCCCGGTTCCGGCACGG 5340

Sbjct 5281 ............................................................ 5340

Query 5341 CGGGCCATTACAATGCCTGTCCCAGCCCCTAGGGTTCGCAAGGTCGTTACCGAACCTCAA 5400

Sbjct 5341 ............................................................ 5400

Query 5401 CCGGAGCCAGAAGCTCCTATCCCGGCACCAAGAAAGAGAAAAACCATCAGCACCACACCT 5460

Sbjct 5401 .TA..A..G................................................... 5460

Query 5461 CCGCATAACCCCGGGGACTTTGTTCCCAGGGTACCGGTTGAATTACCGTGGGAGCCGGAA 5520

Sbjct 5461 ............................................................ 5520

Query 5521 GACCTAGACATCCAATTCGGTGATTTGGAGCCACGCCGGCGGAACACCAGGGACCGGGAT 5580

Sbjct 5521 ............................................................ 5580

Query 5581 GTCAGCACAGGAATACAGTTCGGTGACATCGACTTTAACCAGTCCTGACTAGGCAGGGCT 5640

Sbjct 5581 ............................................................ 5640

Query 5641 GGCGCGTATATCTTTTCGTCTGACACTGGCCCAGGTCACCTACAGCAGAAGTCCGTAAGG 5700

Sbjct 5641 ............................................................ 5700

Query 5701 CAGCATGAATTGCCATGCGAGACTCTGTATGCCCATGAAGACGAACGCATATACCCGCCG 5760

Sbjct 5701 ............................................................ 5760

Query 5761 GCATTTGACGGAGAGAAAGAGAAGGTACTCCAGGCAAAGATGCAGATGGCCCCGACAGAA 5820

Sbjct 5761 ............................................................ 5820

Query 5821 GCGAATAAGAGCAGGTACCAGTCGAGGAAAGTAGAGAACATGAAGGCATTAATTGTAGAA 5880

Sbjct 5821 ............................................................ 5880

Query 5881 AGACTACGCGAAGGAGCAAAATTGTACCTCCATGAGCAAACCGACAAGGTACCTACGTAC 5940

Sbjct 5881 ............................................................ 5940

Query 5941 ACCAGCAAGTACCCTAGACCTGTGTACTCACCATCGGTGGATGACAGCCTGAGCGATCCG 6000

Sbjct 5941 ............................................................ 6000

Query 6001 GACGTGGCCGTGGCCGCTTGTAACTCTTTCTTAGAAGAGAATTACCCGACCGTGGCGAAC 6060

Sbjct 6001 ............................................................ 6060

Query 6061 TACCAGATAACTGACGAGTATGACGCCTATCTGGACTTGGTCGACGGCTCTGAAAGCTGC 6120

Sbjct 6061 ............................................................ 6120

Query 6121 CTCGACAGAGCTACGTTCTGCCCGGCCAAACTAAGATGTTACCCTAAGCACCATGCATAC 6180

Sbjct 6121 ............................................................ 6180

Query 6181 CACCGACCACAAATCAGGAGCGCAGTACCTTCCCCATTCCAAAACACGCTACAAAACGTG 6240

Sbjct 6181 ............................................................ 6240

Query 6241 CTAGCCGCGGCCACTAAAAGAAACTGCAATGTCACCCAAATGAGAGAATTACCAACCATG 6300

Sbjct 6241 ............................................................ 6300

Query 6301 GACTCTGCGGTGTTCAACGTAGAAAGCTTCAAAAAGTACGCCTGTACCGGCGAATATTGG 6360

Sbjct 6301 ............................................................ 6360

Query 6361 CAAGAATTTAAAGACAACCCCATACGGATCACCACCGAAAACATAACGACGTACGTGGCT 6420

Sbjct 6361 ............................................................ 6420

Query 6421 AAACTCAAGGGCCCGAAGGCTGCTGCTCTTTTTGCCAAGACGCATAACCTGGTGCCGCTC 6480

Sbjct 6421 ............................................................ 6480

Query 6481 CAGGAGGTGCCAATGGACCGCTTCGTGATGGACATGAAGAGAGACGTGAAAGTTACACCA 6540

Sbjct 6481 ............................................................ 6540

Query 6541 GGTACCAAGCATACTGAAGAAAGGCCCAAAGTGCAAGTGATTCAAGCGGCGGAACCATTG 6600

Sbjct 6541 ............................................................ 6600

Query 6601 GCCACAGCATATCTATGCGGGATCCACAGAGAGTTAGTCAGGCGGTTGAAAGCCGTTTTG 6660

Sbjct 6601 ............................................................ 6660

Query 6661 ACCCCGAACATCCACACTCTGTTTGACATGTCGGCGGAGGACTTTGATGCCATCATAGCG 6720

Sbjct 6661 ............................................................ 6720

Query 6721 GCACATTTCCAACCGGGAGATGCTGTACTGGAGACAGATATCGCATCCTTCGATAAGAGC 6780

Sbjct 6721 ............................................................ 6780

Query 6781 CAAGACGACTCCTTAGCGCTAACGGCGCTGATGCTTCTGGAAGACCTCGGGGTCGACCAA 6840

Sbjct 6781 ............................................................ 6840

Query 6841 GAACTGCTGGACCTTATCGAAGCCGCGTTCGGCGAGATCACGAGTGTGCATCTACCTACC 6900

Sbjct 6841 ............................................................ 6900

Query 6901 GGTACAAGATTTAAATTCGGTGCTATGATGAAGTCAGGAATGTTTCTCACACTCTTCATC 6960

Sbjct 6901 ............................................................ 6960

Query 6961 AATACGCTGCTGAACATTGTCATAGCGTGCCGCGTTTTACGCGACAAACTATCGTCCTCG 7020

Sbjct 6961 ............................................................ 7020

Query 7021 GCCTGCGCCGCCTTCATAGGTGATGACAACATAGTGCATGGTGTGAGGTCAGACCCGCTA 7080

Sbjct 7021 ............................................................ 7080

Query 7081 ATGGCAGAAAGGTGTGCGAGTTGGGTCAACATGGAAGTAAAGATCATCGATGCCACAATG 7140

Sbjct 7081 ............................................................ 7140

Query 7141 TGTGAGAAACCACCGTACTTTTGTGGAGGATTCATCCTGTACGACAGTGTCACCGGTACA 7200

Sbjct 7141 ............................................................ 7200

Query 7201 GCGTGTAGGGTTGCAGACCCACTAAAGAGGCTGTTCAAACTCGGAAAACCACTACCAGCG 7260

Sbjct 7201 ............................................................ 7260

Query 7261 GATGACAACCAGGATGAAGACAGAAGAAGGGCACTAAAAGACGAAACAGTTAAGTGGTCC 7320

Sbjct 7261 ............................................................ 7320

Query 7321 CGCATCGGATTGAGAGAAGAATTAGACGTGGCACTGAGCTCAAGATACCAAGTCAGTGGT 7380

Sbjct 7321 ............................................................ 7380

Query 7381 GTCGGAAACATCACTAGAGCGATGTCCACGTTGTCTAAGAACCTGAAGTCTTTTAGGAAA 7440

Sbjct 7381 ............................................................ 7440

Query 7441 ATAAGAGGTCCCATTGTACATCTGTACGGCGGTCCTAAATAGATGCAGGAGTACACTACA 7500

Sbjct 7441 ............................................................ 7500

Query 7501 TCTAAAGACCACGCATTATAGACACCATGAATTACATCCCATCTCAAACCTTTTACGGAC 7560

Sbjct 7501 ............................................................ 7560

Query 7561 GCCGTTGGCGACCACGCCCGGCGTTCCGTCCGTGGCGGGTGCCGTTGCAGCCGGCCCCAC 7620

Sbjct 7561 ............................................................ 7620

Query 7621 CCATGATGATTCCTGAGCTGCAAACTCCGATCGTCCAGGCCCAACAGATGCAGCAACTAA 7680

Sbjct 7621 ............................................................ 7680

Query 7681 TCAGTGCAGTTTCTGCCCTTACGACTaagcaaaatggtaaagcaccgaagaagtcgaaga 7740

Sbjct 7681 ............................................................ 7740

Query 7741 aaaagccgcaaaaaacgaagactaagaaaaacgaacagcaaaagaaaaacgagaataaga 7800

Sbjct 7741 ............................................................ 7800

Query 7801 aacCACCACCCAAGCAGAAGAATCCGGCTAAGAAGAAGAAACCAGGAAAAAGGGAACGAA 7860

Sbjct 7801 ............................................................ 7860

Query 7861 TGTGCATGAAGATAGAAAACGATTGCATCTTCGAGGTCAAGCTCGACGGTAAGGTCACGG 7920

Sbjct 7861 ............................................................ 7920

Query 7921 GATATGCATGCCTAGTCGGGGATAAAGTGATGAAGCCGGCACACGTCAAAGGTGTGATCG 7980

Sbjct 7921 ............................................................ 7980

Query 7981 ACAACCCCGATCTAGCGAAGCTTACCTACAAGAAATCGAGCAAGTATGACCTAGAGTGCG 8040

Sbjct 7981 ............................................................ 8040

Query 8041 CCCAGATACCAGTGCACATGAAGTCAGATGCTTCAAAATACACCCACGAAAAACCAGAAG 8100

Sbjct 8041 ............................................................ 8100

Query 8101 GGCACTACAATTGGCATCACGGTGCAGTGCAGTACAGTGGCGGCAGGTTCACAATCCCGA 8160

Sbjct 8101 ............................................................ 8160

Query 8161 CAGGCGCAGGTAAACCAGGAGACAGCGGCCGGCCGATCTTCGATAACAAAGGACGTGTGG 8220

Sbjct 8161 ............................................................ 8220

Query 8221 TGGCCATTGTCCTGGGAGGGGCCAACGAAGGAGCTAGAACTGCCCTATCTGTCGTGACCT 8280

Sbjct 8221 ............................................................ 8280

Query 8281 GGACCAAAGACATGGTCACACGGTATACCCCAGAAGGAACTGAAGAATGGTCCGCCGCCT 8340

Sbjct 8281 ............................................................ 8340

Query 8341 TGATGATGTGTGTTTTAGCCAACGTTACGTTCCCATGCTCAGAGCCCGCATGTGCACCCT 8400

Sbjct 8341 ............................................................ 8400

Query 8401 GTTGCTATGAAAAACAACCAGAACAGACACTGAGGATGTTGGAGGACAACGTGGACCGCC 8460

Sbjct 8401 ............................................................ 8460

Query 8461 CGGGCTACTACGATCTGCTCGAGGCCACGATGACATGTAACAATAGTGCACGCCACCGTC 8520

Sbjct 8461 ............................................................ 8520

Query 8521 GCAGTGTGACGGAACACTTCAATGTCTATAAGGCCACGAAACCGTACCTAGCGTACTGCG 8580

Sbjct 8521 ............................................................ 8580

Query 8581 CGGATTGCGGAGACGGGCAGTCCTGCTACAGCCCGGTGGCTATAGAAAAAATTAGGGATG 8640

Sbjct 8581 ............................................................ 8640

Query 8641 AGGCTTCTGATGGCATGATAAAGATCCAGGTCGCAGCGCAAATTGGTATCAATAAAGGAG 8700

Sbjct 8641 ............................................................ 8700

Query 8701 GAACACACGAACACAACAAAATTAGGTACATCGCCGGGCATGACATGAAAGAGGCTAACC 8760

Sbjct 8701 ............................................................ 8760

Query 8761 GGGATTCTTTGCAAGTGCATACATCTGGTACGTGCGCTATTCGAGGCACGATGGGCCACT 8820

Sbjct 8761 ............................................................ 8820

Query 8821 TCATCGCGGCCTACTGCCCTCCAGGGGACGAATTAAAGGTCCAGTTCCAAGATGCAGAAT 8880

Sbjct 8821 ............................................................ 8880

Query 8881 CGCACATCCAGGCTTGCAAAGTGCAGTACAAACACGCTCCGGCCCCGGTAGGCAGAGAAA 8940

Sbjct 8881 ............................................................ 8940

Query 8941 AATTCACCGTCAGGCCTCACTTCGGCATCGAAGTGCCATGCACAACGTACCAGCTGACTA 9000

Sbjct 8941 ............................................................ 9000

Query 9001 CCGCACCGACGGAGGAAGAGATCGACATGCATACCCCACCGGATATCCCAGACATAACGT 9060

Sbjct 9001 ............................................................ 9060

Query 9061 TGCTGTCGCAGCAGTCAGGTAATGTAAAGATCACAGCAGGAGGAAAAACCATCAGATACA 9120

Sbjct 9061 ............................................................ 9120

Query 9121 ACTGTACGTGTGGGAGTGGCAACGTGGGCACAACCAGTAGTGACAAGACTATCAATTCGT 9180

Sbjct 9121 ............................................................ 9180

Query 9181 GCAAAATTGCACAGTGCCACGCTGCGGTGACTAACCACGACAAGTGGCAGTACACCTCCT 9240

Sbjct 9181 ............................................................ 9240

Query 9241 CGTTTGTCCCTAGAGCCGACCAGTTGTCTCGCAAAGGTAAAGTGCACGTACCCTTCCCTT 9300

Sbjct 9241 ............................................................ 9300

Query 9301 TGACCGACTCCACATGCAGGGTGCCCCTTGCACGTGCACCGGGTGTCACATACGGAAAGA 9360

Sbjct 9301 ............................................................ 9360

Query 9361 GAGAACTGACAGTGAAACTGCATCCAGACCATCCCACACTGTTGACGTACCGGAGTCTAG 9420

Sbjct 9361 ............................................................ 9420

Query 9421 GAGCAGATCCCCGCCCGTATGAGGAGTGGATAGACCGATACGCCGAACGGACCATACCGG 9480

Sbjct 9421 ............................................................ 9480

Query 9481 TGACTGAAGAGGGGATCGAGTACAGATGGGGAAACAACCCGCCCGTACGCTTGTGGGCCC 9540

Sbjct 9481 ............................................................ 9540

Query 9541 AACTGACAACTGAAGGCAAACCCCATGGGTGGCCGCACGAGATCATACTCTACTACTACG 9600

Sbjct 9541 ............................................................ 9600

Query 9601 GGCTATACCCAGCAGCCACCATCGCCGCCGTCTCCGCCGCGGGTCTCGCAGTCGTACTAT 9660

Sbjct 9601 ............................................................ 9660

Query 9661 CACTGCTGGCGTCATGCTACATGTTCGCCACCGCACGCCGCAAGTGCTTGACCCCTTACG 9720

Sbjct 9661 .......................................................A.... 9720

Query 9721 CCCTGACCCCCGGAGCCATCGTCCCGGTAACACTGGGAGTACTATGCTGCGCACCGCGAG 9780

Sbjct 9721 ............................................................ 9780

Query 9781 CGCATGCCGCGTCGTTTGCGGAATCTATGGCGTATTTATGGGATGAGAACCAAACCCTAT 9840

Sbjct 9781 ............................................................ 9840

Query 9841 TTTGGCTGGAGCTTGCAACGCCGCTCGCTGCCATAATCATACTTGTATGCTGCCTGAAGA 9900

Sbjct 9841 ............................................................ 9900

Query 9901 ACTTGCTTTGCTGCTGCAAACCGCTTTCTTTTTTAGTGTTGGTGAGCCTGGGAACTCCCG 9960

Sbjct 9901 ............................................................ 9960

Query 9961 TCGTAAAATCTTACGAACACACCGCAACGATCCCGAATGTGGTGGGATTCCCGTATAAGG 10020

Sbjct 9961 ............................................................ 10020

Query 10021 CTCACATTGAGAGGAACGGCTTCTCCCCGATGACCCTACAGCTTGAGGTACTTGGAACCA 10080

Sbjct 10021 ............................................................ 10080

Query 10081 GTTTGGAACCCACACTAAACTTAGAGTATATAACCTGTGAATACAAGACAGTCGTGCCGT 10140

Sbjct 10081 ............................................................ 10140

Query 10141 CACCTTACATCAAGTGTTGCGGGACATCAGAATGCAGATCCAAGGAGCGCCCCGACTATC 10200

Sbjct 10141 ............................................................ 10200

Query 10201 AATGCCAGGTCTACACAGGAGTGTACCCATTCATGTGGGGCGGCGCATACTGCTTCTGCG 10260

Sbjct 10201 ............................................................ 10260

Query 10261 ACACTGAGAACACCCAGTTGAGTGAAGCATACGTTGACAGATCAGACGTATGCAAGCATG 10320

Sbjct 10261 ............................................................ 10320

Query 10321 ATCATGCCGCGGCCTACAAGGCGCACACTGCGGCAATGAAAGCCACCATCCGAATAAGCT 10380

Sbjct 10321 ............................................................ 10380

Query 10381 ACGGGAACCTCAACCAGACAACGACAGCGTTCGTCAACGGGGAGCACACAGTGAACGTCG 10440

Sbjct 10381 ............................................................ 10440

Query 10441 GGGGCAGCAGGTTTACTTTTGGCCCGATCTCCACTGCCTGGACGCCTTTCGACAACAAGA 10500

Sbjct 10441 ............................................................ 10500

Query 10501 TTGCCGTCTACAAGAACGATGTCTACAACCAGGACTTCCCACCCTATGGGTCTGGACAAC 10560

Sbjct 10501 ............................................................ 10560

Query 10561 CAGGGAGGTTTGGAGACATCCAGAGCAGGACTGTAGAGAGCAAAGACCTGTATGCCAATA 10620

Sbjct 10561 ............................................................ 10620

Query 10621 CCGCCCTCAAGTTGTCAAGACCTTCGTCCGGTACTGTTCATGTGCCTTATACACAGACCC 10680

Sbjct 10621 ............................................................ 10680

Query 10681 CTTCCGGCTTTAAGTACTGGATAAAAGAGAGAGGCACATCGCTGAATGACAAGGCTCCTT 10740

Sbjct 10681 ............................................................ 10740

Query 10741 TTGGGTGCATAATCAAGACCAACCCAGTCAGAGCTGAAAATTGTGCCGTTGGCAACATCC 10800

Sbjct 10741 ............................................................ 10800

Query 10801 CAGTCTCCATGGACATTCCGGACTCCGCGTTTACGCGCGTGATTGACGCACCTGCCGTCA 10860

Sbjct 10801 ............................................................ 10860

Query 10861 CAAACCTGGAGTGCCAAGTGGCGGTTTGCACGCACTCATCGGACTTCGGCGGGATTGCGA 10920

Sbjct 10861 ............................................................ 10920

Query 10921 CTCTGACCTTCAAAACTGACAAACCCGGAAAATGTGCTGTCCACTCTCATTCGAACGTAG 10980

Sbjct 10921 ............................................................ 10980

Query 10981 CTACCATACAGGAGGCAGCTGTGGACATCAAAACAGATGGCAAGATAACCCTGCATTTCT 11040

Sbjct 10981 ............................................................ 11040

Query 11041 CTACAGCATCAGCTTCCCCGGCATTCAAGGTATCTGTGTGCAGTGCCAAAACGACATGCA 11100

Sbjct 11041 ............................................................ 11100

Query 11101 CGGCAGCGTGTGAGCCGCCGAAGGATCATATCGTCCCTTACGGAGCGAGCCATAACAACC 11160

Sbjct 11101 ............................................................ 11160

Query 11161 AAGTTTTTCCAGACATGTCGGGTACGGCAATGACATGGGTGCAGCGGGTAGCTGGCGGAC 11220

Sbjct 11161 ............................................................ 11220

Query 11221 TCGGCGGGCTGACACTCGCTGCAGTGGCAGTACTCATACTGGTGACGTGTGTGACCATGC 11280

Sbjct 11221 ............................................................ 11280

Query 11281 GCCGCTAACCAGGAGGCTTGACACAACGTACATATATAAGCATTATAGTTCTAATAAAGC 11340

Sbjct 11281 ............................................................ 11340

Query 11341 ACATAAATAACCAAGTAGATCAAAGGGCTAACTAACCCCTGAATAGTAACAAAACACAAA 11400

Sbjct 11341 ..............................C.A........................... 11400

Query 11401 ATACAAAAACATTAGTTCAAAGGGCTAGTAACCCCTGAATAGTAACAAAACATAAAAACT 11460

Sbjct 11401 ............................................................ 11460

Query 11461 AAAAACAGTAGTTCAAAGGGCTATACAACCCCTGAATAGTAACaaaacacagaaaaacca 11520

Sbjct 11461 ............................................................ 11520

Query 11521 taaaaatcttaaaaataaataatCTGATCATCTAAATTTTACTAATTGGAAATAGCCGAA 11580

Sbjct 11521 ..............T............................................. 11580

Query 11581 CTCTACGGAGACGTAGGCGTCCGAACTCCACGGAGACGTAGGACAAAATTCTGCCGAACC 11640

Sbjct 11581 ............................................................ 11640

Query 11641 CCAGACCA 11648

Sbjct 11641 ........ 11648

**Query N544** Length: 11686

>Getah virus strain MM2021, complete genome

Sequence ID: MN849355.1 Length: 11690

Range 1: 4 to 11686

Score:21525 bits(11656), Expect:0.0,

Identities:11674/11683(99%), Gaps:0/11683(0%), Strand: Plus/Plus

Query 4 GCGGACGTGTGACATCACCGTTCGCTCTTTCTAGGATCCTTTGCTACTCCACATAGTGAG 63

Sbjct 4 ............................................................ 63

Query 64 AGACAGACAACCCAAATGAAGGTAACCGTGGACGTTGAGGCTGACAGCCCATTCCTTAAG 123

Sbjct 64 ............................................................ 123

Query 124 GCCCTTCAGAAGGCGTTTCCCGCCTTTGAGGTTGAATCACAGCAGGTCACACCGAATGAC 183

Sbjct 124 ............................................................ 183

Query 184 CATGCTAACGCCAGAGCATTTTCGCATCTGGCTACCAAACTGATTGAGCAAGAGGTTCCA 243

Sbjct 184 ............................................................ 243

Query 244 ACAGGCGTCACCATCCTGGATGTGGGTAGTGCACCCGCAAGGAGGTTGATGTCTGACCAC 303

Sbjct 244 ............................................................ 303

Query 304 ACCTACCACTGCATCTGCCCTATGAAAAGTGCGGAAGACCCAGAGAGGTTGGCGAATTAC 363

Sbjct 304 ............................................................ 363

Query 364 GCTCGAAAGCTGGCGAAAGCATCGGGGACTGTGCTAGACAAGAATGTGTCCGGGAAGATA 423

Sbjct 364 ............................................................ 423

Query 424 ACGGACCTTCAAGACGTCATGGCCACTCCAGACTTGGAATCCCCGACTTTTTGCCTGCAC 483

Sbjct 424 ............................................................ 483

Query 484 ACCGACGAGACGTGCCGCACTAGGGCTGAGGTCGCTGTGTACCAGGACGTATACGCTGTG 543

Sbjct 484 ............................................................ 543

Query 544 CACGCACCGACGTCTCTGTACCACCAGGCAATTAAAGGTGTAAGGACGGCGTATTGGATT 603

Sbjct 544 ............................................................ 603

Query 604 GGATTCGACACCACTCCATTCATGTTCGAGGCACTAGCGGGCGCGTACCCTGCATACTCG 663

Sbjct 604 ............................................................ 663

Query 664 ACCAATTGGGCAGATGAGCAAGTGCTGCAGGCTCGTAACATCGGCCTGTGCGCGACAGGC 723

Sbjct 664 ............................................................ 723

Query 724 CTCTCTGAGGGGCGTCGCGGCAAACTCTCCATCATGAGAAAGAAGTGCTTGCGACCGAGC 783

Sbjct 724 ............................................................ 783

Query 784 GACAGAGTAATGTTTTCGGTCGGGTCCACCTTGTACACCGAGAGCCGAAAGCTGCTGCGC 843

Sbjct 784 ............................................................ 843

Query 844 AGCTGGCATTTACCTTCCGTGTTTCACCTGAAGGGTAAGAACAGTTTTACCTGCAGGTGT 903

Sbjct 844 ............................................................ 903

Query 904 GACACGGTGGTGTCATGCGAAGGTTATGTGGTAAAGAAGATCACCATAAGCCCGGGCATA 963

Sbjct 904 ............................................................ 963

Query 964 TATGGAAAAACAGTTGACTACGCAGTTACCCATCACGCAGAGGGTTTCCTGATGTGCAAG 1023

Sbjct 964 ............................................................ 1023

Query 1024 ATCACTGATACAGTCAGAGGAGAAAGAGTCTCTTTCCCGGTCTGTACCTATGTGCCTGCA 1083

Sbjct 1024 ............................................................ 1083

Query 1084 ACCATATGCGACCAGATGACGGGTATACTCGCCACCGACGTGACACCGGAGGATGCCCAG 1143

Sbjct 1084 ............................................................ 1143

Query 1144 AAGCTCCTGGTCGGATTGAACCAACGCATAGTGGTGAATGGTAGGACGCAAAGAAACACA 1203

Sbjct 1144 ............................................................ 1203

Query 1204 AATACAATGAAAAACTACCTACTGCCAGTGGTAGCGCAAGCATTCAGTAAATGGGCGCGT 1263

Sbjct 1204 ............................................................ 1263

Query 1264 GAGGCGCGCGCAGACATGGAGGATGAAAAACCCCTAGGCACCAGAGAACGCACGTTGACG 1323

Sbjct 1264 ............................................................ 1323

Query 1324 TGTTGTTGCCTGTGGGCGTTTAAAAGCCACAAAATCCACACCATGTATAAGCGGCCTGAA 1383

Sbjct 1324 ............................................................ 1383

Query 1384 ACGCAGACTATCGTCAAAGTGCCTTCTACTTTTGACTCCTTTGTGATACCGAGCCTGTGG 1443

Sbjct 1384 ............................................................ 1443

Query 1444 TCATCCAGCCTTTCCATGGGTATCAGACAGAGGATCGAACTGCTTCTCAGCGCAAGAATG 1503

Sbjct 1444 ............................................................ 1503

Query 1504 GCCCAAAGCCTACCATACTCAGGAGACCGCACTGAAGCTCGAGCGGCTGAAGAAGAAGAG 1563

Sbjct 1504 ............................................................ 1563

Query 1564 AAAGAGGCGCAGGAGGCAGAACTTACGAGGGCAGCGCTGCCACCACTAGTGAGTGGCTCT 1623

Sbjct 1564 ............................................................ 1623

Query 1624 TGCGCTGACGATATCACCCAGGTAGATGTAGAGGAATTAACCTTCAGAGCCGGAGCCGGG 1683

Sbjct 1624 ............................................................ 1683

Query 1684 GTTGTGGAAACACCCAGGAACGCCCTGAAGGTCACACCGCAAGCACACGACCACCTCATA 1743

Sbjct 1684 ............................................................ 1743

Query 1744 GGCTCCTACTTGATCCTTTCCCCCCAAACGGTCTTGAAAAGTGAGAAGCTGGCACCCATC 1803

Sbjct 1744 ............................................................ 1803

Query 1804 CATCCTCTTGCCGAGCAAGTCACGGTCATGACCCACTCTGGGAGATCCGGCAGATACCCA 1863

Sbjct 1804 ............................................................ 1863

Query 1864 GTCGACAAGTACGACGGACGGGTATTGATCCCAACAGGAGCGGCCATCCCAGTGAGTGAG 1923

Sbjct 1864 ............................................................ 1923

Query 1924 TTCCAGGCACTCAGCGAGAGCGCAACCATGGTGTACAATGAGAGGGAATTTATAAATCGT 1983

Sbjct 1924 ............................................................ 1983

Query 1984 AAACTACACCACATAGCGCTATACGGGCCAGCCTTGAACACCGACGAGGAAAGCTACGAA 2043

Sbjct 1984 ............................................................ 2043

Query 2044 AAAGTGAGAGCTGAGAGGGCAGAGACAGAGTATGTGTTCGACGTGGACAAGAAGGCATGC 2103

Sbjct 2044 ............................................................ 2103

Query 2104 ATCAAGAAGGAGGAGGCATCAGGCCTTGTGTTAACAGGGGACCTAATCAATCCACCTTTC 2163

Sbjct 2104 ............................................................ 2163

Query 2164 CACGAATTCGCATACGAAGGACTCAAGATCCGCCCAGCAGCCCCGTACCACACGACGATC 2223

Sbjct 2164 ............................................................ 2223

Query 2224 ATTGGGGTGTTTGGCGTTCCAGGTTCGGGCAAGTCGGCTATCATTAAGAACATGGTGACG 2283

Sbjct 2224 ............................................................ 2283

Query 2284 ACCCGCGATCTAGTGGCCAGTGGAAAGAAGGAGAACTGCCAAGAGATCATGAACGACGTA 2343

Sbjct 2284 ............................................................ 2343

Query 2344 AAGAGGCAACGCGGGTTGGATGTGACCGCTAGAACCGTCGACTCAATCTTATTAAATGGG 2403

Sbjct 2344 ............................................................ 2403

Query 2404 TGCAAGAGAGGCGTAGAAAACCTTTACGTCGATGAGGCGTTCGCGTGCCACTCGGGTACT 2463

Sbjct 2404 ............................................................ 2463

Query 2464 TTGCTAGCGCTTATTGCGCTAGTGAGACCGTCAGGTAAGGTAGTACTGTGCGGCGACCCT 2523

Sbjct 2464 ............................................................ 2523

Query 2524 AAGCAGTGTGGCTTCTTCAACTTGATGCAACTGAAGGTGCACTACAACCACAACATCTGT 2583

Sbjct 2524 ............................................................ 2583

Query 2584 ACAAGGGTGCTCCATAAGAGCATCTCCAGAAGATGCACACTACCTGTCACGGCGATCGTG 2643

Sbjct 2584 ............................................................ 2643

Query 2644 TCCACCTTGCACTACCAAGGGAAGATGAGAACGACGAACCGATGCAATACTCCTATTCAG 2703

Sbjct 2644 ............................................................ 2703

Query 2704 ATCGACACCACCGGTTCCTCCAAACCGGCCTCAGGAGATATCGTGTTAACGTGTTTCCGC 2763

Sbjct 2704 ............................................................ 2763

Query 2764 GGCTGGGTGAAACAACTGCAAATCGACTATCGTGGACACGAGGTGATGACCGCAGCTGCT 2823

Sbjct 2764 ............................................................ 2823

Query 2824 TCTCAGGGTCTGACAAGGAAAGGCGTGTACGCCGTGAGACAGAAAGTGAACGAAAACCCA 2883

Sbjct 2824 ............................................................ 2883

Query 2884 CTGTACTCACCTCTGTCAGAGCACGTCAATGTGCTGTTGACCCGAACTGAAAACCGACTG 2943

Sbjct 2884 ............................................................ 2943

Query 2944 GTGTGGAAAACACTGTCGGGTGACCCGTGGATAAAGGTGTTAACCAATGTTCCACGAGGA 3003

Sbjct 2944 ............................................................ 3003

Query 3004 GATTTCAGTGCGACTTTGGAGGAATGGCAAGAAGAACATGAAGGTATCATGAGAGTGTTG 3063

Sbjct 3004 ............................................................ 3063

Query 3064 AACGAGCGACCAGCGGAGGTTGATCCATTCCAAAATAAGGCCAAAGTGTGCTGGGCAAAA 3123

Sbjct 3064 ............................................................ 3123

Query 3124 TGTCTGGTGCAAGTTCTTGAGACAGCCGGAATACGTATGACGGCAGATGAATGGAACACC 3183

Sbjct 3124 ............................................................ 3183

Query 3184 ATCTTGGCTTTTAGAGAAGACAGAGCATACTCACCAGAAGTCGCTCTCAATGAGATCTGC 3243

Sbjct 3184 ............................................................ 3243

Query 3244 ACTCGTTACTACGGCGTCGACCTAGACAGCGGCCTATTCTCAGCGCAGTCAGTCTCCCTC 3303

Sbjct 3244 ............................................................ 3303

Query 3304 TTTTATGAGAACAACCACTGGGACAACAGGCCTGGAGGACGTATGTATGGGTTCAACCAC 3363

Sbjct 3304 ............................................................ 3363

Query 3364 GAAGTAGCCAGGAAATATGCAGCTAGGTTTCCATTTCTACGTGGCAACATGAACTCGGGG 3423

Sbjct 3364 ............................................................ 3423

Query 3424 CTACAACTAAACGTCCCTGAGAGGAAGCTCCAACCTTTTAGCGCTGAATGCAACATAGTA 3483

Sbjct 3424 ............................................................ 3483

Query 3484 CCATCCAATCGCCGGTTGCCGCATGCTCTAGTCACCACTTATCAGCAGTGCCGCGGGGAG 3543

Sbjct 3484 ............................................................ 3543

Query 3544 AGGGTAGAGTGGTTGCTGAAAAAGATCCCTGGCCACCAAATGTTACTTGTAAGCGAGTAC 3603

Sbjct 3544 ............................................................ 3603

Query 3604 AACCTGGTGATACCTCACAAAAGAGTCTTCTGGATTGCACCTCCGAGGGTGTCAGGCGCG 3663

Sbjct 3604 ............................................................ 3663

Query 3664 GACCGCACGTACGACTTGGACCTAGGGTTACCTATGGATGCAGGTCGTTACGATTTGGTA 3723

Sbjct 3664 ............................................................ 3723

Query 3724 TTCGTCAACATCCATACCGAGTACCGGCAGCACCACTACCAACAATGCGTCGACCATTCA 3783

Sbjct 3724 ............................................................ 3783

Query 3784 ATGCGCCTGCAGATGCTGGGAGGGGATTCACTACACCTGCTTAGACCAGGAGGCTCGCTG 3843

Sbjct 3784 ............................................................ 3843

Query 3844 CTGATGAGAGCATATGGTTATGCAGACAGAGTCAGCGAGATGGTGGTGACAGCCCTGGCT 3903

Sbjct 3844 ............................................................ 3903

Query 3904 AGGAAATTCTCGGCGTTCCGTGTCCTGAGGCCAGCGTGTGTGACGAGCAACACAGAAGTG 3963

Sbjct 3904 ............................................................ 3963

Query 3964 TTCCTGCTGTTTTCTAACTTCGATAACGGCAGGAGAGCGGTAACCTTGCACCAAGCTAAT 4023

Sbjct 3964 ............................................................ 4023

Query 4024 CAGAGACTTAGCTCGATGTATGCCTGCAACGGACTGCACACTGCAGGCTGTGCACCGTCA 4083

Sbjct 4024 ............................................................ 4083

Query 4084 TACAGGGTTCGCCGCGCTGATATATCAGGACACGGTGAGGAAGCGGTCGTAAATGCTGCC 4143

Sbjct 4084 ............................................................ 4143

Query 4144 AACGCCAAAGGTACCGTGAGCGATGGAGTGTGTAGGGCGGTCGCTAAGAAGTGGCCATCA 4203

Sbjct 4144 ............................................................ 4203

Query 4204 TCATTCAAAGGGGCTGCAACTCCAGTCGGCACAGCCAAGATGATCCGCGCTGATGGCATG 4263

Sbjct 4204 ............................................................ 4263

Query 4264 ACCGTAATCCACGCAGTGGGACCGAATTTCTCCACCGTAACAGAAGCTGAAGGGGACAGA 4323

Sbjct 4264 ............................................................ 4323

Query 4324 GAGCTAGCGGCCGCGTACCGAGCTGTGGCTAGCATAATTAGTACTAACAACATAAAGAGC 4383

Sbjct 4324 ............................................................ 4383

Query 4384 GTCGCAGTACCGCTGCTGTCCACAGGCACCTTCTCCGGCGGTAAGGACAGAGTGATGCAG 4443

Sbjct 4384 ............................................................ 4443

Query 4444 TCCTTGAACCACTTATTCACGGCGTTGGATGCCACCGATGCAGACGTGGTTATCTACTGC 4503

Sbjct 4444 ............................................................ 4503

Query 4504 AGAGACAAAAACTGGGAAAAGAAGATTCAGGAAGCCATCGACAGGCGGACGGCAATAGAG 4563

Sbjct 4504 ............................................................ 4563

Query 4564 CTCGTATCTGAAGACGTGACTTTGGAAACCGACCTGGTTAGGGTACACCCGGATAGTTGC 4623

Sbjct 4564 ................................T........................... 4623

Query 4624 TTAGTCGGCAGAAACGGTTACAGCGCAACTGACGGTAAGCTATACTCTTACCTTGAGGGC 4683

Sbjct 4624 ............................................................ 4683

Query 4684 ACGAGGTTTCACCAGACGGCGGTCGACATGGCTGAAATATCAACTTTATGGCCACGACTC 4743

Sbjct 4684 ............................................................ 4743

Query 4744 CAAGACGCTAACGAGCAGATCTGCCTGTACGCCCTAGGGGAGACGATGGACAGCATACGC 4803

Sbjct 4744 ............................................................ 4803

Query 4804 ACCAAATGCCCAGTAGAAGACGCCGATTCGTCTTCGCCGCCGAAAACGGTACCATGTTTA 4863

Sbjct 4804 .................................A.......................... 4863

Query 4864 TGTCGGTATGCGATGACCGCAGAGCGAGTTGCCAGACTTAGGATGAACAACACCAAAAAC 4923

Sbjct 4864 ............................................................ 4923

Query 4924 ATCATCGTGTGCTCCTCCTTTCCATTGCCGAAGTACAGGATAGAAGGCGTGCAGAAGGTG 4983

Sbjct 4924 ............................................................ 4983

Query 4984 AAGTGTGACCGAGTGCTAATTTTTGATCAGACCGTCCCGTCACTAGTAAGTCCCAGAAAG 5043

Sbjct 4984 ............................................................ 5043

Query 5044 TACATACAGCAGCTGCCGGAACAGCTGGATAATGTGAGCCTGACTTCTGCGACGTCGACG 5103

Sbjct 5044 ............................................................ 5103

Query 5104 GGATCCGCTTGGTCGTTCCCATCGGAAACGACCTACGAAACTATGGAAGTCGTAGCCGAG 5163

Sbjct 5104 ............................................................ 5163

Query 5164 GTACACACCGAACCTCCGATCCCTCCGCCTCGTAGACGTAGAGCAGCCGTCGTCCAACTT 5223

Sbjct 5164 ............................................................ 5223

Query 5224 AGACAAGACCTGGAAGTCACCGAGGAGATCGAACCGTACGCGATACAGCAAGCAGAGATC 5283

Sbjct 5224 ............................................................ 5283

Query 5284 ATGGTCATGGAGAGGGTCGCGACGACAGATATACGCGCTATCCCGGTTCCGGCACGGCGG 5343

Sbjct 5284 ............................................................ 5343

Query 5344 GCCATTACAATGCCTGTCCCAGCCCCTAGGGTTCGCAAGGTCGTTACCGAACCTCAACCG 5403

Sbjct 5344 ..........................................................TA 5403

Query 5404 GAGCCAGAAGCTCCTATCCCGGCACCAAGAAAGAGAAAAACCATCAGCACCACACCTCCG 5463

Sbjct 5404 ..A..G...................................................... 5463

Query 5464 CATAACCCCGGGGACTTTGTTCCCAGGGTACCGGTTGAATTACCGTGGGAGCCGGAAGAC 5523

Sbjct 5464 ............................................................ 5523

Query 5524 CTAGACATCCAATTCGGTGATTTGGAGCCACGCCGGCGGAACACCAGGGACCGGGATGTC 5583

Sbjct 5524 ............................................................ 5583

Query 5584 AGCACAGGAATACAGTTCGGTGACATCGACTTTAACCAGTCCTGACTAGGCAGGGCTGGC 5643

Sbjct 5584 ............................................................ 5643

Query 5644 GCGTATATCTTTTCGTCTGACACTGGCCCAGGTCACCTACAGCAGAAGTCCGTAAGGCAG 5703

Sbjct 5644 ............................................................ 5703

Query 5704 CATGAATTGCCATGCGAGACTCTGTATGCCCATGAAGACGAACGCATATACCCGCCGGCA 5763

Sbjct 5704 ............................................................ 5763

Query 5764 TTTGACGGAGAGAAAGAGAAGGTACTCCAGGCAAAGATGCAGATGGCCCCGACAGAAGCG 5823

Sbjct 5764 ............................................................ 5823

Query 5824 AATAAGAGCAGGTACCAGTCGAGGAAAGTAGAGAACATGAAGGCATTAATTGTAGAAAGA 5883

Sbjct 5824 ............................................................ 5883

Query 5884 CTACGCGAAGGAGCAAAATTGTACCTCCATGAGCAAACCGACAAGGTACCTACGTACACC 5943

Sbjct 5884 ............................................................ 5943

Query 5944 AGCAAGTACCCTAGACCTGTGTACTCACCATCGGTGGATGACAGCCTGAGCGATCCGGAC 6003

Sbjct 5944 ............................................................ 6003

Query 6004 GTGGCCGTGGCCGCTTGTAACTCTTTCTTAGAAGAGAATTACCCGACCGTGGCGAACTAC 6063

Sbjct 6004 ............................................................ 6063

Query 6064 CAGATAACTGACGAGTATGACGCCTATCTGGACTTGGTCGACGGCTCTGAAAGCTGCCTC 6123

Sbjct 6064 ............................................................ 6123

Query 6124 GACAGAGCTACGTTCTGCCCGGCCAAACTAAGATGTTACCCTAAGCACCATGCATACCAC 6183

Sbjct 6124 ............................................................ 6183

Query 6184 CGACCACAAATCAGGAGCGCAGTACCTTCCCCATTCCAAAACACGCTACAAAACGTGCTA 6243

Sbjct 6184 ............................................................ 6243

Query 6244 GCCGCGGCCACTAAAAGAAACTGCAATGTCACCCAAATGAGAGAATTACCAACCATGGAC 6303

Sbjct 6244 ............................................................ 6303

Query 6304 TCTGCGGTGTTCAACGTAGAAAGCTTCAAAAAGTACGCCTGTACCGGCGAATATTGGCAA 6363

Sbjct 6304 ............................................................ 6363

Query 6364 GAATTTAAAGACAACCCCATACGGATCACCACCGAAAACATAACGACGTACGTGGCTAAA 6423

Sbjct 6364 ............................................................ 6423

Query 6424 CTCAAGGGCCCGAAGGCTGCTGCTCTTTTTGCCAAGACGCATAACCTGGTGCCGCTCCAG 6483

Sbjct 6424 ............................................................ 6483

Query 6484 GAGGTGCCAATGGACCGCTTCGTGATGGACATGAAGAGAGACGTGAAAGTTACACCAGGT 6543

Sbjct 6484 ............................................................ 6543

Query 6544 ACCAAGCATACTGAAGAAAGGCCCAAAGTGCAAGTGATTCAAGCGGCGGAACCATTGGCC 6603

Sbjct 6544 ............................................................ 6603

Query 6604 ACAGCATATCTATGCGGGATCCACAGAGAGTTAGTCAGGCGGTTGAAAGCCGTTTTGACC 6663

Sbjct 6604 ............................................................ 6663

Query 6664 CCGAACATCCACACTCTGTTTGACATGTCGGCGGAGGACTTTGATGCCATCATAGCGGCA 6723

Sbjct 6664 ............................................................ 6723

Query 6724 CATTTCCAACCGGGAGATGCTGTACTGGAGACAGATATCGCATCCTTCGATAAGAGCCAA 6783

Sbjct 6724 ............................................................ 6783

Query 6784 GACGACTCCTTAGCGCTAACGGCGCTGATGCTTCTGGAAGACCTCGGGGTCGACCAAGAA 6843

Sbjct 6784 ............................................................ 6843

Query 6844 CTGCTGGACCTTATCGAAGCCGCGTTCGGCGAGATCACGAGTGTGCATCTACCTACCGGT 6903

Sbjct 6844 ............................................................ 6903

Query 6904 ACAAGATTTAAATTCGGTGCTATGATGAAGTCAGGAATGTTTCTCACACTCTTCATCAAT 6963

Sbjct 6904 ............................................................ 6963

Query 6964 ACGCTGCTGAACATTGTCATAGCGTGCCGCGTTTTACGCGACAAACTATCGTCCTCGGCC 7023

Sbjct 6964 ............................................................ 7023

Query 7024 TGCGCCGCCTTCATAGGTGATGACAACATAGTGCATGGTGTGAGGTCAGACCCGCTAATG 7083

Sbjct 7024 ............................................................ 7083

Query 7084 GCAGAAAGGTGTGCGAGTTGGGTCAACATGGAAGTAAAGATCATCGATGCCACAATGTGT 7143

Sbjct 7084 ............................................................ 7143

Query 7144 GAGAAACCACCGTACTTTTGTGGAGGATTCATCCTGTACGACAGTGTCACCGGTACAGCG 7203

Sbjct 7144 ............................................................ 7203

Query 7204 TGTAGGGTTGCAGACCCACTAAAGAGGCTGTTCAAACTCGGAAAACCACTACCAGCGGAT 7263

Sbjct 7204 ............................................................ 7263

Query 7264 GACAACCAGGATGAAGACAGAAGAAGGGCACTAAAAGACGAAACAGTTAAGTGGTCCCGC 7323

Sbjct 7264 ............................................................ 7323

Query 7324 ATCGGATTGAGAGAAGAATTAGACGTGGCACTGAGCTCAAGATACCAAGTCAGTGGTGTC 7383

Sbjct 7324 ............................................................ 7383

Query 7384 GGAAACATCACTAGAGCGATGTCCACGTTGTCTAAGAACCTGAAGTCTTTTAGGAAAATA 7443

Sbjct 7384 ............................................................ 7443

Query 7444 AGAGGTCCCATTGTACATCTGTACGGCGGTCCTAAATAGATGCAGGAGTACACTACATCT 7503

Sbjct 7444 ............................................................ 7503

Query 7504 AAAGACCACGCATTATAGACACCATGAATTACATCCCATCTCAAACCTTTTACGGACGCC 7563

Sbjct 7504 ............................................................ 7563

Query 7564 GTTGGCGACCACGCCCGGCGTTCCGTCCGTGGCGGGTGCCGTTGCAGCCGGCCCCACCCA 7623

Sbjct 7564 ............................................................ 7623

Query 7624 TGATGATTCCTGAGCTGCAAACTCCGATCGTCCAGGCCCAACAGATGCAGCAACTAATCA 7683

Sbjct 7624 ............................................................ 7683

Query 7684 GTGCAGTTTCTGCCCTTACGACTaagcaaaatggtaaagcaccgaagaagtcgaagaaaa 7743

Sbjct 7684 ............................................................ 7743

Query 7744 agccgcaaaaaacgaagactaagaaaaacgaacagcaaaagaaaaacgagaataagaaac 7803

Sbjct 7744 ............................................................ 7803

Query 7804 CACCACCCAAGCAGAAGAATCCGGCTAAGAAGAAGAAACCAGGAAAAAGGGAACGAATGT 7863

Sbjct 7804 ............................................................ 7863

Query 7864 GCATGAAGATAGAAAACGATTGCATCTTCGAGGTCAAGCTCGACGGTAAGGTCACGGGAT 7923

Sbjct 7864 ............................................................ 7923

Query 7924 ATGCATGCCTAGTCGGGGATAAAGTGATGAAGCCGGCACACGTCAAAGGTGTGATCGACA 7983

Sbjct 7924 ............................................................ 7983

Query 7984 ACCCCGATCTAGCGAAGCTTACCTACAAGAAATCGAGCAAGTATGACCTAGAGTGCGCCC 8043

Sbjct 7984 ............................................................ 8043

Query 8044 AGATACCAGTGCACATGAAGTCAGATGCTTCAAAATACACCCACGAAAAACCAGAAGGGC 8103

Sbjct 8044 ............................................................ 8103

Query 8104 ACTACAATTGGCATCACGGTGCAGTGCAGTACAGTGGCGGCAGGTTCACAATCCCGACAG 8163

Sbjct 8104 ............................................................ 8163

Query 8164 GCGCAGGTAAACCAGGAGACAGCGGCCGGCCGATCTTCGATAACAAAGGACGTGTGGTGG 8223

Sbjct 8164 ............................................................ 8223

Query 8224 CCATTGTCCTGGGAGGGGCCAACGAAGGAGCTAGAACTGCCCTATCTGTCGTGACCTGGA 8283

Sbjct 8224 ............................................................ 8283

Query 8284 CCAAAGACATGGTCACACGGTATACCCCAGAAGGAACTGAAGAATGGTCCGCCGCCTTGA 8343

Sbjct 8284 ............................................................ 8343

Query 8344 TGATGTGTGTTTTAGCCAACGTTACGTTCCCATGCTCAGAGCCCGCATGTGCACCCTGTT 8403

Sbjct 8344 ............................................................ 8403

Query 8404 GCTATGAAAAACAACCAGAACAGACACTGAGGATGTTGGAGGACAACGTGGACCGCCCGG 8463

Sbjct 8404 ............................................................ 8463

Query 8464 GCTACTACGATCTGCTCGAGGCCACGATGACATGTAACAATAGTGCACGCCACCGTCGCA 8523

Sbjct 8464 ............................................................ 8523

Query 8524 GTGTGACGGAACACTTCAATGTCTATAAGGCCACGAAACCGTACCTAGCGTACTGCGCGG 8583

Sbjct 8524 ............................................................ 8583

Query 8584 ATTGCGGAGACGGGCAGTCCTGCTACAGCCCGGTGGCTATAGAAAAAATTAGGGATGAGG 8643

Sbjct 8584 ............................................................ 8643

Query 8644 CTTCTGATGGCATGATAAAGATCCAGGTCGCAGCGCAAATTGGTATCAATAAAGGAGGAA 8703

Sbjct 8644 ............................................................ 8703

Query 8704 CACACGAACACAACAAAATTAGGTACATCGCCGGGCATGACATGAAAGAGGCTAACCGGG 8763

Sbjct 8704 ............................................................ 8763

Query 8764 ATTCTTTGCAAGTGCATACATCTGGTACGTGCGCTATTCGAGGCACGATGGGCCACTTCA 8823

Sbjct 8764 ............................................................ 8823

Query 8824 TCGCGGCCTACTGCCCTCCAGGGGACGAATTAAAGGTCCAGTTCCAAGATGCAGAATCGC 8883

Sbjct 8824 ............................................................ 8883

Query 8884 ACATCCAGGCTTGCAAAGTGCAGTACAAACACGCTCCGGCCCCGGTAGGCAGAGAAAAAT 8943

Sbjct 8884 ............................................................ 8943

Query 8944 TCACCGTCAGGCCTCACTTCGGCATCGAAGTGCCATGCACAACGTACCAGCTGACTACCG 9003

Sbjct 8944 ............................................................ 9003

Query 9004 CACCGACGGAGGAAGAGATCGACATGCATACCCCACCGGATATCCCAGACATAACGTTGC 9063

Sbjct 9004 ............................................................ 9063

Query 9064 TGTCGCAGCAGTCAGGTAATGTAAAGATCACAGCAGGAGGAAAAACCATCAGATACAACT 9123

Sbjct 9064 ............................................................ 9123

Query 9124 GTACGTGTGGGAGTGGCAACGTGGGCACAACCAGTAGTGACAAGACTATCAATTCGTGCA 9183

Sbjct 9124 ............................................................ 9183

Query 9184 AAATTGCACAGTGCCACGCTGCGGTGACTAACCACGACAAGTGGCAGTACACCTCCTCGT 9243

Sbjct 9184 ............................................................ 9243

Query 9244 TTGTCCCTAGAGCCGACCAGTTGTCTCGCAAAGGTAAAGTGCACGTACCCTTCCCTTTGA 9303

Sbjct 9244 ............................................................ 9303

Query 9304 CCGACTCCACATGCAGGGTGCCCCTTGCACGTGCACCGGGTGTCACATACGGAAAGAGAG 9363

Sbjct 9304 ............................................................ 9363

Query 9364 AACTGACAGTGAAACTGCATCCAGACCATCCCACACTGTTGACGTACCGGAGTCTAGGAG 9423

Sbjct 9364 ............................................................ 9423

Query 9424 CAGATCCCCGCCCGTATGAGGAGTGGATAGACCGATACGCCGAACGGACCATACCGGTGA 9483

Sbjct 9424 ............................................................ 9483

Query 9484 CTGAAGAGGGGATCGAGTACAGATGGGGAAACAACCCGCCCGTACGCTTGTGGGCCCAAC 9543

Sbjct 9484 ............................................................ 9543

Query 9544 TGACAACTGAAGGCAAACCCCATGGGTGGCCGCACGAGATCATACTCTACTACTACGGGC 9603

Sbjct 9544 ............................................................ 9603

Query 9604 TATACCCAGCAGCCACCATCGCCGCCGTCTCCGCCGCGGGTCTCGCAGTCGTACTATCAC 9663

Sbjct 9604 ............................................................ 9663

Query 9664 TGCTGGCGTCATGCTACATGTTCGCCACCGCACGCCGCAAGTGCTTGACCCCTTACGCCC 9723

Sbjct 9664 ....................................................A....... 9723

Query 9724 TGACCCCCGGAGCCATCGTCCCGGTAACACTGGGAGTACTATGCTGCGCACCGCGAGCGC 9783

Sbjct 9724 ............................................................ 9783

Query 9784 ATGCCGCGTCGTTTGCGGAATCTATGGCGTATTTATGGGATGAGAACCAAACCCTATTTT 9843

Sbjct 9784 ............................................................ 9843

Query 9844 GGCTGGAGCTTGCAACGCCGCTCGCTGCCATAATCATACTTGTATGCTGCCTGAAGAACT 9903

Sbjct 9844 ............................................................ 9903

**Figure S2**. Alignments of N554 GETV_MM2021,_ and N544 and GETV_MM2021_

Query 9904 TGCTTTGCTGCTGCAAACCGCTTTCTTTTTTAGTGTTGGTGAGCCTGGGAACTCCCGTCG 9963

Sbjct 9904 ............................................................ 9963

Query 9964 TAAAATCTTACGAACACACCGCAACGATCCCGAATGTGGTGGGATTCCCGTATAAGGCTC 10023

Sbjct 9964 ............................................................ 10023

Query 10024 ACATTGAGAGGAACGGCTTCTCCCCGATGACCCTACAGCTTGAGGTACTTGGAACCAGTT 10083

Sbjct 10024 ............................................................ 10083

Query 10084 TGGAACCCACACTAAACTTAGAGTATATAACCTGTGAATACAAGACAGTCGTGCCGTCAC 10143

Sbjct 10084 ............................................................ 10143

Query 10144 CTTACATCAAGTGTTGCGGGACATCAGAATGCAGATCCAAGGAGCGCCCCGACTATCAAT 10203

Sbjct 10144 ............................................................ 10203

Query 10204 GCCAGGTCTACACAGGAGTGTACCCATTCATGTGGGGCGGCGCATACTGCTTCTGCGACA 10263

Sbjct 10204 ............................................................ 10263

Query 10264 CTGAGAACACCCAGTTGAGTGAAGCATACGTTGACAGATCAGACGTATGCAAGCATGATC 10323

Sbjct 10264 ............................................................ 10323

Query 10324 ATGCCGCGGCCTACAAGGCGCACACTGCGGCAATGAAAGCCACCATCCGAATAAGCTACG 10383

Sbjct 10324 ............................................................ 10383

Query 10384 GGAACCTCAACCAGACAACGACAGCGTTCGTCAACGGGGAGCACACAGTGAACGTCGGGG 10443

Sbjct 10384 ............................................................ 10443

Query 10444 GCAGCAGGTTTACTTTTGGCCCGATCTCCACTGCCTGGACGCCTTTCGACAACAAGATTG 10503

Sbjct 10444 ............................................................ 10503

Query 10504 CCGTCTACAAGAACGATGTCTACAACCAGGACTTCCCACCCTATGGGTCTGGACAACCAG 10563

Sbjct 10504 ............................................................ 10563

Query 10564 GGAGGTTTGGAGACATCCAGAGCAGGACTGTAGAGAGCAAAGACCTGTATGCCAATACCG 10623

Sbjct 10564 ............................................................ 10623

Query 10624 CCCTCAAGTTGTCAAGACCTTCGTCCGGTACTGTTCATGTGCCTTATACACAGACCCCTT 10683

Sbjct 10624 ............................................................ 10683

Query 10684 CCGGCTTTAAGTACTGGATAAAAGAGAGAGGCACATCGCTGAATGACAAGGCTCCTTTTG 10743

Sbjct 10684 ............................................................ 10743

Query 10744 GGTGCATAATCAAGACCAACCCAGTCAGAGCTGAAAATTGTGCCGTTGGCAACATCCCAG 10803

Sbjct 10744 ............................................................ 10803

Query 10804 TCTCCATGGACATTCCGGACTCCGCGTTTACGCGCGTGATTGACGCACCTGCCGTCACAA 10863

Sbjct 10804 ............................................................ 10863

Query 10864 ACCTGGAGTGCCAAGTGGCGGTTTGCACGCACTCATCGGACTTCGGCGGGATTGCGACTC 10923

Sbjct 10864 ............................................................ 10923

Query 10924 TGACCTTCAAAACTGACAAACCCGGAAAATGTGCTGTCCACTCTCATTCGAACGTAGCTA 10983

Sbjct 10924 ............................................................ 10983

Query 10984 CCATACAGGAGGCAGCTGTGGACATCAAAACAGATGGCAAGATAACCCTGCATTTCTCTA 11043

Sbjct 10984 ............................................................ 11043

Query 11044 CAGCATCAGCTTCCCCGGCATTCAAGGTATCTGTGTGCAGTGCCAAAACGACATGCACGG 11103

Sbjct 11044 ............................................................ 11103

Query 11104 CAGCGTGTGAGCCGCCGAAGGATCATATCGTCCCTTACGGAGCGAGCCATAACAACCAAG 11163

Sbjct 11104 ............................................................ 11163

Query 11164 TTTTTCCAGACATGTCGGGTACGGCAATGACATGGGTGCAGCGGGTAGCTGGCGGACTCG 11223

Sbjct 11164 ............................................................ 11223

Query 11224 GCGGGCTGACACTCGCTGCAGTGGCAGTACTCATACTGGTGACGTGTGTGACCATGCGCC 11283

Sbjct 11224 ............................................................ 11283

Query 11284 GCTAACCAGGAGGCTTGACACAACGTACATATATAAGCATTATAGTTCTAATAAAGCACA 11343

Sbjct 11284 ............................................................ 11343

Query 11344 TAAATAACCAAGTAGATCAAAGGGCTAACTAACCCCTGAATAGTAACAAAACACAAAATA 11403

Sbjct 11344 ...........................C.A.............................. 11403

Query 11404 CAAAAACATTAGTTCAAAGGGCTAGTAACCCCTGAATAGTAACAAAACATAAAAACTAAA 11463

Sbjct 11404 ............................................................ 11463

Query 11464 AACAGTAGTTCAAAGGGCTATACAACCCCTGAATAGTAACAAAACACAGAAAAACCATAA 11523

Sbjct 11464 ............................................................ 11523

Query 11524 AAATCTTAAAATTAAATAATCTGATCATCTAAATTTTACTAATTGGAAATAGCCGAACTC 11583

Sbjct 11524 ............................................................ 11583

Query 11584 TACGGAGACGTAGGCGTCCGAACTCCACGGAGACGTAGGACAAAATTCTGCCGAACCCCA 11643

Sbjct 11584 ............................................................ 11643

Query 11644 GACCACCGGGGACGTAGGCGTCTAATTTGtttttttAATATTT 11686

Sbjct 11644 ........................................... 11686


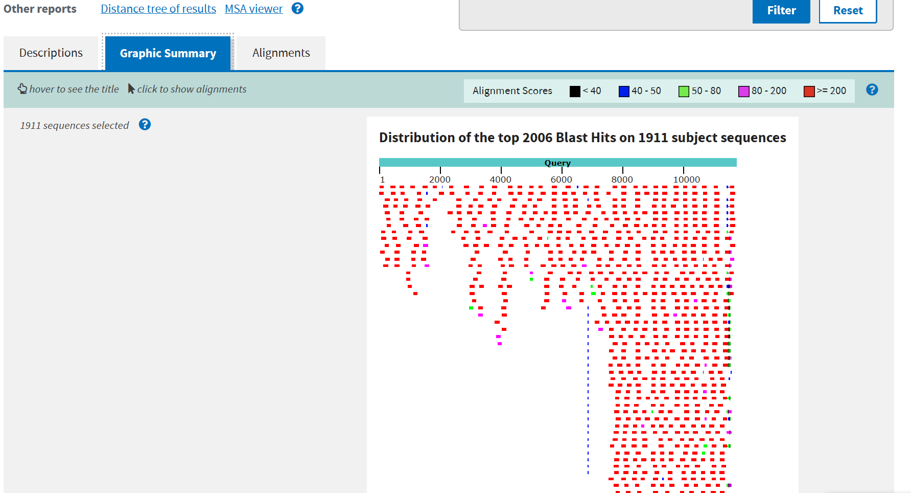


GETV_MM2021_ genome

Encoding the

structural polyprotein

(Capid-E3-E2-6k-E1)

Encoding the non-structural polyprotein

(nsP1-nsP2-nsP3-nsP4)

**A**

**C**


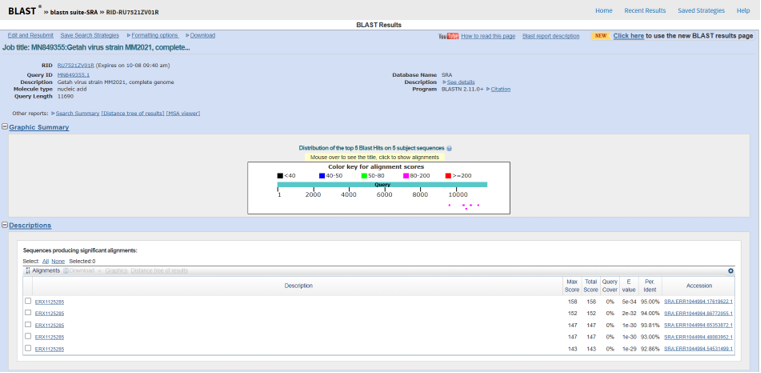


GETV_MM2021_ genome

**E value % Identity**

5e-34 95%

2e-33 94%

1e-30 93.81%

1e-30 93%

1e-29 92.66%

**Figure S3**. BLASTn read alignments to GETV and RRV genomes. (**A**) GETV reads from PRJNA561663 aligned to GETV. GETV k-mer mining of SRA Accessions SRR10014823, SRR10014824, SRR10014825, SRR10014826 from BioProject PRJNA561663 indicated the presence of GETV sequences. BLASTn searches identified reads in the aforementioned high-throughput sequencing data sets that mapped to the GETV genome. During infection subgenomic RNA encoding the structural polyproteins (right) is usually more abundant than subgenomic RNA encoding the non-structural polyproteins (left) (Wilson et al. PLoS Pathog 2017, 13, e1006155), suggesting these reads were obtained from replicating GETV; (**B**) GETV reads from SRR1745767 aligned to the GETV genome using BLASTn; (**C**) GETV reads from ERR1044994 aligned to the GETV genome using BLASTn; (**D**) RRV reads from SRR11094162 aligned to the RRV genome using BLASTn.


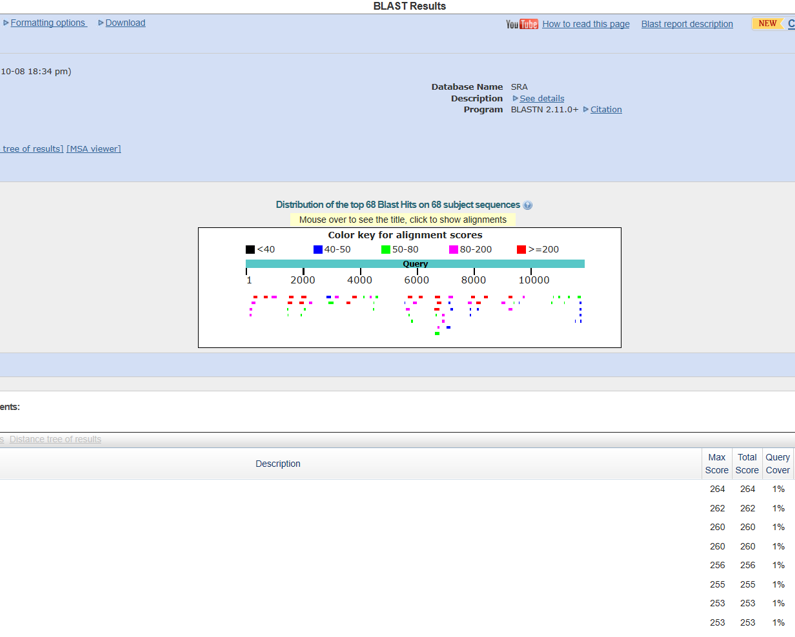


**D**

RRV genome


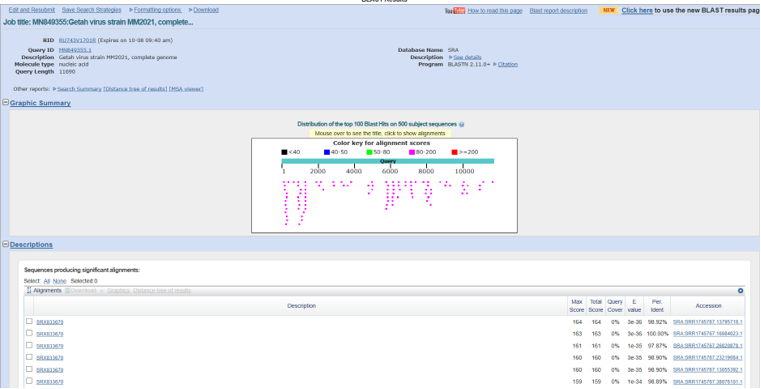


GETV_MM2021_ genome

**B**


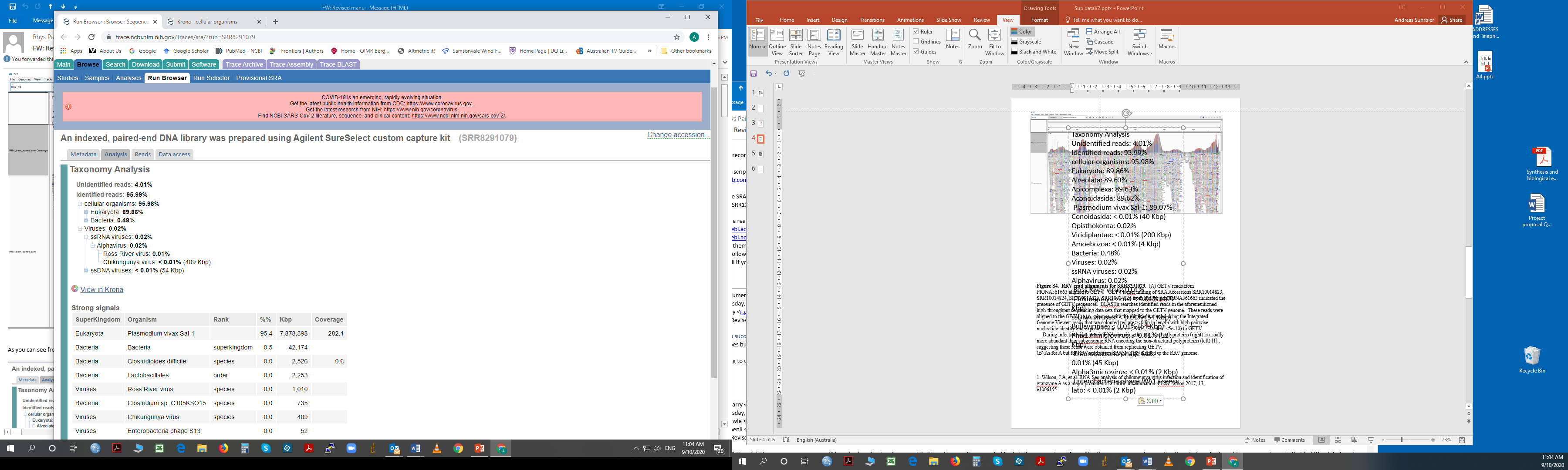


**A**

**B**


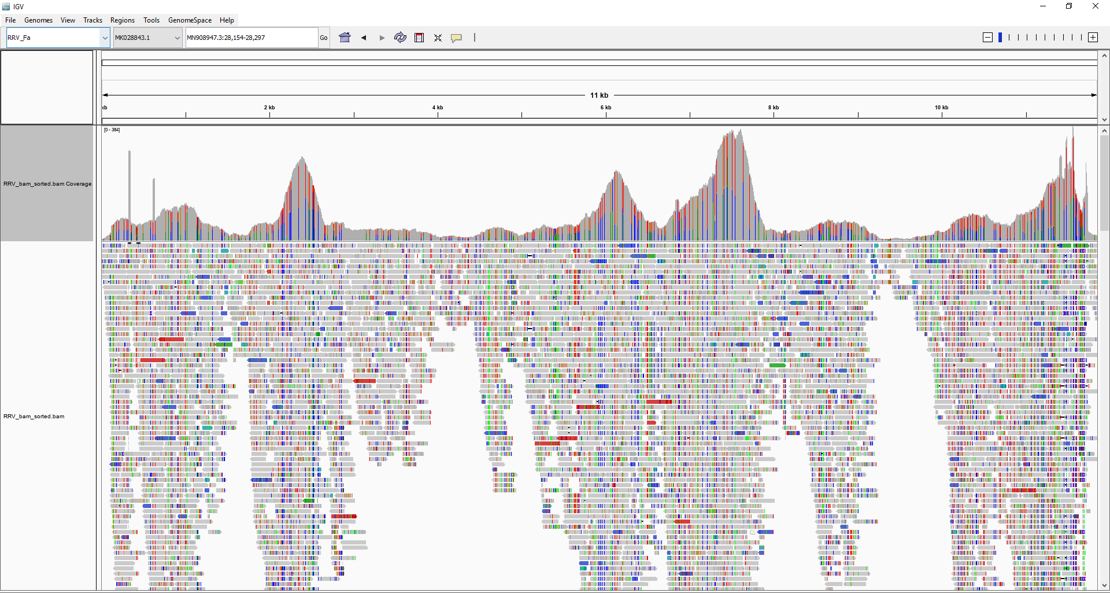


**C**

Ross River virus strain T48. Sequence ID: DQ226993.1

Query 5110 GCACGCAGATACCGTGAGTTTGGATTCTACAGTATCTACAGGATCTGCGTGGTCATTCCC 5169

|||||||||||||||||| ||||||||||||||||| |||||||| ||||||||||||||

Sbjct 5067 GCACGCAGATACCGTGAGCTTGGATTCTACAGTATCCACAGGATCCGCGTGGTCATTCCC 5126

Query 5170 ATCTGAGGCCACGTATGAGACCATGGAAGTAGTAGCAGAGGTGCACCRCTCGGAACCACC 5229

||||||||||||||||||||||||||||||||||||||||||||||| ||||||||||||

Sbjct 5127 ATCTGAGGCCACGTATGAGACCATGGAAGTAGTAGCAGAGGTGCACCACTCGGAACCACC 5186

Query 5230 AGTCCCACCACCGCGCAGGCGTCGTGCGCAGGTGACGATGCACCACCAGGAGCTGTTGGA 5289

|||||| |||||||||||||||||||||||||||||||||||||||||||||||||||||

Sbjct 5187 AGTCCCGCCACCGCGCAGGCGTCGTGCGCAGGTGACGATGCACCACCAGGAGCTGTTGGA 5246

Query 5290 AGTCTCYGACATGCACACCCCGATTGCGGCAAGGGTCGAGATCCCTGYGTACGATACCGC 5349

|||||| |||||||||||||||||||||||||||||||||||||| | ||||||||||||

Sbjct 5247 AGTCTCTGACATGCACACCCCGATTGCGGCAAGGGTCGAGATCCCCGTGTACGATACCGC 5306

Query 5350 TGTTGTAGTGGAGAGAGTGGCAATTCCTTGCAYAAGCGAGTATGCAAMMCCMATACCAGC 5409

|||||||||||||||||||||||||||||||| |||||||||||||| || ||||||||

Sbjct 5307 TGTTGTAGTGGAGAGAGTGGCAATTCCTTGCACAAGCGAGTATGCAAAACCCATACCAGC 5366

Query 5410 ACCRCRSGCAGCAAGGGTCGTWCCCGTGCCGGCACCRCGCATTCAGCGAGCGTCGACGTA 5469

||| | |||||||||||||| |||||||||||||| |||||||||||||||||||||||

Sbjct 5367 ACCACGGGCAGCAAGGGTCGTACCCGTGCCGGCACCACGCATTCAGCGAGCGTCGACGTA 5426

Query 5470 CAGAGTCTCTCCTAYACCCACGCCTCGCGTTCTGAGAGCCTCGGTATGCAGTGTGACCAC 5529

|||||||||||||| |||||||||||||||||||||||||||||||||||||||||||||

Sbjct 5427 CAGAGTCTCTCCTACACCCACGCCTCGCGTTCTGAGAGCCTCGGTATGCAGTGTGACCAC 5486

Query 5530 TAGCGCTGGGGTAGAGTTCCCTTGGGCRCCTGAAGATCTGGAGGTACTYACCGAGCCTGT 5589

||||||||||||||||||||||||||| |||||||||||||||||||| |||||||||||

Sbjct 5487 TAGCGCTGGGGTAGAGTTCCCTTGGGCGCCTGAAGATCTGGAGGTACTCACCGAGCCTGT 5546

Query 5590 GCACTGCRAAATGCGCGAGCCGGTTGAGTTACCGTGGGAGCCKGAGGACRTTGAYATCCA 5649

||||||| |||||||||||||||||||||||||||||||||| |||||| |||| |||||

Sbjct 5547 GCACTGCAAAATGCGCGAGCCGGTTGAGTTACCGTGGGAGCCTGAGGACGTTGATATCCA 5606

Query 5650 RTTCGGAGATTTTGAAACAYCCGACAAAATYCAATTCGGCGAYATYGATTTTGACCAATT 5709

|||||||||||||||||| |||||||||| ||||||||||| || ||||||||||||||

Sbjct 5607 GTTCGGAGATTTTGAAACATCCGACAAAATCCAATTCGGCGATATTGATTTTGACCAATT 5666

Query 5710 CTGACTAGGCAGAGCGGGGGCGTACATCTTCTCGTCTGATACCGGACCRGGGCACTTACA 5769

|||||||||||||||||||||||||||||||||||||||||||||||| |||||||||||

Sbjct 5667 CTGACTAGGCAGAGCGGGGGCGTACATCTTCTCGTCTGATACCGGACCAGGGCACTTACA 5726

Query 5770 ACAGAAGTCAGTACGGCAACACGCACTACCRTGCGAAATGCTATACGYCCACGAGGAAGA 5829

|||||||||||||||||||||||||||||| |||||||||||||||| ||||||||||||

Sbjct 5727 ACAGAAGTCAGTACGGCAACACGCACTACCGTGCGAAATGCTATACGTCCACGAGGAAGA 5786

Query 5830 ACGGACGTAcccccccGCACTGGATGAGGCCAGGGAGAAACTGCTGCWGGCAAAAATGCA 5889

||||||||||||||||||||||||||||||||||||||||||||||| ||||||||||||

Sbjct 5787 ACGGACGTACCCCCCCGCACTGGATGAGGCCAGGGAGAAACTGCTGCAGGCAAAAATGCA 5846

Query 5890 GATGGCACCTACGGAAGCAAACAAGAGYAGGTACCAATCAAGGAAGGTTGAAAAYATGAA 5949

||||||||||||||||||||||||||| |||||||||||||||||||||||||| |||||

Sbjct 5847 GATGGCACCTACGGAAGCAAACAAGAGCAGGTACCAATCAAGGAAGGTTGAAAACATGAA 5906

Query 5950 GGCAGTGATYATAGATAGGCTGAAGGATGGAGCAAGAAYCTACCTGACAGAACAGTCAGA 6009

||||||||| |||||||||||||||||||||||||||| |||||||||||||||||||||

Sbjct 5907 GGCAGTGATCATAGATAGGCTGAAGGATGGAGCAAGAACCTACCTGACAGAACAGTCAGA 5966

Query 6010 GAAGATTCCAACCTATGTTAGTAAGTACCCGCGGCCAGTTTACTCGCCGTCGGTRGAGGA 6069

|||||||||||||||||||||||||||||||||||||||||||||||||||||| |||||

Sbjct 5967 GAAGATTCCAACCTATGTTAGTAAGTACCCGCGGCCAGTTTACTCGCCGTCGGTAGAGGA 6026

Query 6070 TAGCTTGCAGAATCCMGAGGTCGCTGTGGCGGCCTGCAATGCTTTCCTGGAAGCCAATTA 6129

||||||||||||||| ||||||||||||||||||||||||||||||||||||||||||||

Sbjct 6027 TAGCTTGCAGAATCCCGAGGTCGCTGTGGCGGCCTGCAATGCTTTCCTGGAAGCCAATTA 6086

Query 6130 CCCGACAGTGGCTAGTTACCAGATCACGGACGAGTATGATGCCTACTTGGATATGGTTGA 6189

||||||||||||||||||||||||||||||||||||||||||||||||||||||||||||

Sbjct 6087 CCCGACAGTGGCTAGTTACCAGATCACGGACGAGTATGATGCCTACTTGGATATGGTTGA 6146

Query 6190 TGGGTCAGAGAGYTGYTTAGACCGGGCWACCTTCTGCCCGGCAAAATTACGCTGCTACCC 6249

|||||||||||| || ||||||||||| ||||||||||||||||||||||||||||||||

Sbjct 6147 TGGGTCAGAGAGTTGTTTAGACCGGGCAACCTTCTGCCCGGCAAAATTACGCTGCTACCC 6206

Query 6250 AAAGCATCATGCTTACCACCAACCGCAGGTTAGGAGCGCGGTCCCATCACCATTTCAAAA 6309

||||||||||||||||||||||||||||||||||||||||||||||||||||||||||||

Sbjct 6207 AAAGCATCATGCTTACCACCAACCGCAGGTTAGGAGCGCGGTCCCATCACCATTTCAAAA 6266

Query 6310 CACCYTGCAGAATGTGCTAGCAGCAGCCACGAAGAGAAACTGCAATGTTACACAGATGAG 6369

|||| |||||||||||||||||||||||||||||||||||||||||||||||||||||||

Sbjct 6267 CACCCTGCAGAATGTGCTAGCAGCAGCCACGAAGAGAAACTGCAATGTTACACAGATGAG 6326

Query 6370 AGAGCTACCCACTYTAGACTCAGCCGTGYTTAACGTGGAATGCTTCAAAAAATTCGCATG 6429

||||||||||||| |||||||||||||| |||||||||||||||||||||||||||||||

Sbjct 6327 AGAGCTACCCACTCTAGACTCAGCCGTGCTTAACGTGGAATGCTTCAAAAAATTCGCATG 6386

Query 6430 CAACGGAGAATACTGGCAGGAATTCAAAGACAACCCAATAAGAATAACTACAGAGAACAT 6489

||||||||||||||||||||||||||||||||||||||||||||||||||||||||||||

Sbjct 6387 CAACGGAGAATACTGGCAGGAATTCAAAGACAACCCAATAAGAATAACTACAGAGAACAT 6446

Query 6490 AACAACKTATGTYACTAGGCTTAAGGGCCCTAAAGCRGCRGCGCTGTTTGCRAAGACTCA 6549

|||||| ||||| ||||||||||||||||||||||| || ||||||||||| ||||||||

Sbjct 6447 AACAACTTATGTTACTAGGCTTAAGGGCCCTAAAGCAGCGGCGCTGTTTGCAAAGACTCA 6506

Query 6550 CAAYYTAGTCCCGCTGCAGGAGGTGCCCATGGAYCGGTTTGTGGTAGATATGAAGAGAGA 6609

||| |||||||||||||||||||||||||||| ||||||||||||||||||||||||||

Sbjct 6507 CAATCTAGTCCCGCTGCAGGAGGTGCCCATGGACCGGTTTGTGGTAGATATGAAGAGAGA 6566

Query 6610 CGTGAAAGTTACCCCTGGCACYAAACATACCGAGGAACGCCCAAAGGTGCAAGTCATCCA 6669

||||||||||||||||||||| ||||||||||||||||||||||||||||||||||||||

Sbjct 6567 CGTGAAAGTTACCCCTGGCACCAAACATACCGAGGAACGCCCAAAGGTGCAAGTCATCCA 6626

Query 6670 GGCYGCCGAACCTTTAGCTACAGCTTATTTATGYGGCATTCACAGGGAGTTRGTCCGCCG 6729

||| ||||||||||||||||||||||||||||| ||||||||||||||||| ||||||||

Sbjct 6627 GGCCGCCGAACCTTTAGCTACAGCTTATTTATGTGGCATTCACAGGGAGTTAGTCCGCCG 6686

Query 6730 CCTGAAGGCCGTCCTGGCCCCGAACATACATACATTGTTCGATATGTCGGCAGAAGATTT 6789

||||||||||||||||||||||||||||||||||||||||||||||||||||||||||||

Sbjct 6687 CCTGAAGGCCGTCCTGGCCCCGAACATACATACATTGTTCGATATGTCGGCAGAAGATTT 6746

Query 6790 TGATGCCATCATAGCTGCACATTTCCAACCAGGCGACGCAGTTTTGGAAACGGACATAGC 6849

||||||||||||||||||||||||||||||||||||||||||||||||||||||||||||

Sbjct 6747 TGATGCCATCATAGCTGCACATTTCCAACCAGGCGACGCAGTTTTGGAAACGGACATAGC 6806

Query 6850 CTCCTTTGACAAGAGCCAAGATGACTCTCTGGCGTTGACGGCACTGATGCTGTTGGAAGA 6909

||||||||||||||||||||||||||||||||||||||||||||||||||||||||||||

Sbjct 6807 CTCCTTTGACAAGAGCCAAGATGACTCTCTGGCGTTGACGGCACTGATGCTGTTGGAAGA 6866

Query 6910 CCTCGGGGTTGACCAAGAACTACTAGACTTGATAGAGGCAGCGTTCGGGGAAATTACCAG 6969

||||||||||||||||||||||||||||||||||||||||||||||||||||||||||||

Sbjct 6867 CCTCGGGGTTGACCAAGAACTACTAGACTTGATAGAGGCAGCGTTCGGGGAAATTACCAG 6926

Query 6970 CGTCCACCTGCCAACAGGTACGCGGTTCAAGTTTGGCGCCATGATGAAGTCCGGAATGTT 7029

||||||||||||||||||||||||||||||||||||||||||||||||||||||||||||

Sbjct 6927 CGTCCACCTGCCAACAGGTACGCGGTTCAAGTTTGGCGCCATGATGAAGTCCGGAATGTT 6986

Query 7030 CCTGACACTGTTTGTAAATACCCTGTTAAACATTGTCATAGCATGCCGTGTACTGCGTGA 7089

||||||||||||||||||||||||||||||||||||||||||||||||||||||||||||

Sbjct 6987 CCTGACACTGTTTGTAAATACCCTGTTAAACATTGTCATAGCATGCCGTGTACTGCGTGA 7046

Query 7090 GAAGCTGACAAACTCCGTCTGCGCCGCGTTTATCGGGGATGACAACATAGTGCACGGGGT 7149

||||||||||||||||||||||||||||||||||||||||||||||||||||||||||||

Sbjct 7047 GAAGCTGACAAACTCCGTCTGCGCCGCGTTTATCGGGGATGACAACATAGTGCACGGGGT 7106

Query 7150 AAGATCCGACCCGTTGATGGCTGAAAGGTGCGCCAGCTGGGTTAATATGGAGGTAAAGAT 7209

||||||||||||||||||||||||||||||||||||||||||||||||||||||||||||

Sbjct 7107 AAGATCCGACCCGTTGATGGCTGAAAGGTGCGCCAGCTGGGTTAATATGGAGGTAAAGAT 7166

Query 7210 AATTGACGCTACCATGTGCGAGAAACCACCATATTTCTGCGGCGGGTTTATATTGTATGA 7269

||||||||||||||||||||||||||||||||||||||||||||||||||||||||||||

Sbjct 7167 AATTGACGCTACCATGTGCGAGAAACCACCATATTTCTGCGGCGGGTTTATATTGTATGA 7226

Query 7270 CAAAGTCACCGGMTCGGCGTGCCGAGTGGCCGACCCTCTGAAAAGGTTATTTAAAYTAGG 7329

|||||||||||| |||||||||||||||||||||||||||||||||||||||||| ||||

Sbjct 7227 CAAAGTCACCGGATCGGCGTGCCGAGTGGCCGACCCTCTGAAAAGGTTATTTAAACTAGG 7286

Query 7330 TAAACCTTTACCCGCCGGAGACACCCAAGATGAAGATCGTAGGCGTGCATTGAAGGATGA 7389

||||||||||||||||||||||||||||||||||||||||||||||||||||||||||||

Sbjct 7287 TAAACCTTTACCCGCCGGAGACACCCAAGATGAAGATCGTAGGCGTGCATTGAAGGATGA 7346

Query 7390 GACGGATAGRTGGGCACGAGTAGGGYTGAAGTCTGAACTGGAAATAGCACTAAGTTCYCG 7449

||||||||| ||||||||||||||| ||||||||||||||||||||||||||||||| ||

Sbjct 7347 GACGGATAGGTGGGCACGAGTAGGGCTGAAGTCTGAACTGGAAATAGCACTAAGTTCTCG 7406

Query 7450 GTATGAGGTGAACGGGACCGGCAACATAGTGCGAGCAATGGCCACACTGGCCAAGAGCYT 7509

|||||||||||||||||||||||||||||||||||||||||||||||||||||||||| |

Sbjct 7407 GTATGAGGTGAACGGGACCGGCAACATAGTGCGAGCAATGGCCACACTGGCCAAGAGCCT 7466

Query 7510 GAAGAATTTTAAAAAGCTGCGTGGACCCATYGTACACCTCTACGGCGGTCCTAAATAGAT 7569

|||||||||||||||||||||||||||||| |||||||||||||||||||||||||||||

Sbjct 7467 GAAGAATTTTAAAAAGCTGCGTGGACCCATCGTACACCTCTACGGCGGTCCTAAATAGAT 7526

Query 7570 GCAGAGACACACCTTCATCTAATACAGCTCACAACAGTAAACATGAATTACATACCAACC 7629

||||||||||||||||||||||||||||||||||||||||||||||||||||||||||||

Sbjct 7527 GCAGAGACACACCTTCATCTAATACAGCTCACAACAGTAAACATGAATTACATACCAACC 7586

Query 7630 CAGACTTTYTACGGACGCCGTTGGCGGCCTCGCCCGGCGTTCCGTCCATGGCAGGTGCCG 7689

|||||||| |||||||||||||||||||||||||||||||||||||||||||||||||||

Sbjct 7587 CAGACTTTTTACGGACGCCGTTGGCGGCCTCGCCCGGCGTTCCGTCCATGGCAGGTGCCG 7646

Query 7690 ATGCAGCCGACACCTACTATGGTTRCACCCATGCTGCAAGCACCAGACCTACAGGCCCAA 7749

|||||||||||||||||||||||| |||||||||||||||||||||||||||||||||||

Sbjct 7647 ATGCAGCCGACACCTACTATGGTTACACCCATGCTGCAAGCACCAGACCTACAGGCCCAA 7706

Query 7750 CAGATGCAACAACTGATCAGCGCTGTCTCTGCAYTAACCACcaaacagaatgtaaaagca 7809

||||||||||||||||||||||||||||||||| ||||||||||||||||||||||||||

Sbjct 7707 CAGATGCAACAACTGATCAGCGCTGTCTCTGCATTAACCACCAAACAGAATGTAAAAGCA 7766

Query 7810 ccaaaagggcaamggaaraagaaacagcagaaaccaaaggaaaagaaggaaaaccagaag 7869

|||||||||||| |||| ||||||||||||||||||||||||||||||||||||||||||

Sbjct 7767 CCAAAAGGGCAACGGAAGAAGAAACAGCAGAAACCAAAGGAAAAGAAGGAAAACCAGAAG 7826

Query 7870 aaaaagccgacgcaaaagaaraagcagcagcagaaaccaaaaccrcaggctaagaagaag 7929

|||||||||||||||||||| ||||||||||||||||||||||| |||||||||||||||

Sbjct 7827 AAAAAGCCGACGCAAAAGAAGAAGCAGCAGCAGAAACCAAAACCACAGGCTAAGAAGAAG 7886

Query 7930 aaaCCAGGGAGAAGAGAAAGAATGTGCATGAAGATCGAGAATGACTGCATATTCGAGGTC 7989

||||||||||||||||||||||||||||||||||||||||||||||||||||||||||||

Sbjct 7887 AAACCAGGGAGAAGAGAAAGAATGTGCATGAAGATCGAGAATGACTGCATATTCGAGGTC 7946

Query 7990 AAACTGGAYGGCAAGGTTACCGGTTATGCGTGCCTAGTCGGAGAYAAGGTCATGAAGCCG 8049

|||||||| ||||||||||||||||||||||||||||||||||| |||||||||||||||

Sbjct 7947 AAACTGGATGGCAAGGTTACCGGTTATGCGTGCCTAGTCGGAGACAAGGTCATGAAGCCG 8006

Query 8050 GCTCACGTTAAAGGCACAATTGATAACCCAGACCTTGCGAAGYTGACTTACAAGAARTCC 8109

|||||||||||||||||||||||||||||||||||||||||| ||||||||||||| |||

Sbjct 8007 GCTCACGTTAAAGGCACAATTGATAACCCAGACCTTGCGAAGCTGACTTACAAGAAATCC 8066

Query 8110 AGTAAGTAYGACCTCGAATGCGCCCAGATMCCAGTGCACATGAAGTCCGACGCCTCCAAG 8169

|||||||| |||||||||||||||||||| ||||||||||||||||||||||||||||||

Sbjct 8067 AGTAAGTATGACCTCGAATGCGCCCAGATACCAGTGCACATGAAGTCCGACGCCTCCAAG 8126

Query 8170 TACACACATGAAAAACCCGAAGGTCATTACAAYTGGCACCATGGAGCAGTGCAGTACAGC 8229

|||||||||||||||||||||||||||||||| |||||||||||||||||||||||||||

Sbjct 8127 TACACACATGAAAAACCCGAAGGTCATTACAATTGGCACCATGGAGCAGTGCAGTACAGC 8186

Query 8230 GGAGGAAGGTTTACCATCCCCACAGGCGCCGGCAAACCRGGAGATAGCGGTAGGCCTATT 8289

|||||||||||||||||||||||||||||||||||||| |||||||||||||||||||||

Sbjct 8187 GGAGGAAGGTTTACCATCCCCACAGGCGCCGGCAAACCGGGAGATAGCGGTAGGCCTATT 8246

Query 8290 TTTGACAACAAAGGGCGAGTRGTGGCCATCGTGTTAGGCGGGGCCAACGAAGGTGCTCGC 8349

|||||||||||||||||||| |||||||||||||||||||||||||||||||||||||||

Sbjct 8247 TTTGACAACAAAGGGCGAGTAGTGGCCATCGTGTTAGGCGGGGCCAACGAAGGTGCTCGC 8306

Query 8350 ACTGCGCTGTCTGTGGTGACGTGGACAAAAGACATGGTCACTCGGGTAACGCCAGAAGGA 8409

||||||||||||||||||||||||||||||||||||||||||||||||||||||||||||

Sbjct 8307 ACTGCGCTGTCTGTGGTGACGTGGACAAAAGACATGGTCACTCGGGTAACGCCAGAAGGA 8366

Query 8410 ACYGAAGAGTGGTCTGCCGCGCTGATGATGTGYATCCTTGCCAACACCTCTTTCCCYTGC 8469

|| ||||||||||||||||||||||||||||| ||||||||||||||||||||||| |||

Sbjct 8367 ACTGAAGAGTGGTCTGCCGCGCTGATGATGTGTATCCTTGCCAACACCTCTTTCCCCTGC 8426

Query 8470 TCATCACCTCCCTGCTACCCCTGCTGCTACGAAAAACAGCCAGAACAGACACTGCGGATG 8529

||||||||||||||||||||||||||||||||||||||||||||||||||||||||||||

Sbjct 8427 TCATCACCTCCCTGCTACCCCTGCTGCTACGAAAAACAGCCAGAACAGACACTGCGGATG 8486

Query 8530 CTGGAAGAYAAYGTGAAYAGACCWGGGTACTATGAGYTACTGGAAGCGTCCATGACATGC 8589

|||||||| || ||||| ||||| |||||||||||| |||||||||||||||||||||||

Sbjct 8487 CTGGAAGACAATGTGAATAGACCAGGGTACTATGAGCTACTGGAAGCGTCCATGACATGC 8546

Query 8590 MGAAACRGATCACGCCACCGCCGTAGTGTAACAGAGCACTTCAATGTGTATAAGGCTACT 8649

||||| |||||||||||||||||||||||||||||||||||||||||||||||||||||

Sbjct 8547 AGAAACAGATCACGCCACCGCCGTAGTGTAACAGAGCACTTCAATGTGTATAAGGCTACT 8606

Query 8650 AGACCATACCTAGCRCATTGCGCTGAYTGCGGGGACGGGTACTTCTGCTATAGCCCAGTT 8709

||||| ||| |||| |||||||||| || ||||||||||||||||||||||||||||||

Sbjct 8607 AGACCGTACTTAGCGTATTGCGCTGACTGTGGGGACGGGTACTTCTGCTATAGCCCAGTT 8666

Query 8710 GCTATCGAGAAGATCCGAGATGAGGCGTCTGATGGCATGCTTAAGATCCAAGTCTCCGCC 8769

|||||||||||||||||||||||||||||||| |||||||| ||||||||||||||||||

Sbjct 8667 GCTATCGAGAAGATCCGAGATGAGGCGTCTGACGGCATGCTCAAGATCCAAGTCTCCGCC 8726

Query 8770 CAAATAGGTCTGGACAAGGCAGGYACCCACGCCCACACGAAGCTCCGATATATGGCTGGT 8829

||||||||||||||||||||||| |||||||||||||||||| |||||||||||||||||

Sbjct 8727 CAAATAGGTCTGGACAAGGCAGGTACCCACGCCCACACGAAGATCCGATATATGGCTGGT 8786

Query 8830 CATGATGTTCAGGAATCTAAGAGAGATTCCTTGAGGGTGTACACGTCCGCAGCGTGYTCY 8889

|||||||||||||||||||||||||||||||||||||||||||||||||||||||| ||

Sbjct 8787 CATGATGTTCAGGAATCTAAGAGAGATTCCTTGAGGGTGTACACGTCCGCAGCGTGCTCT 8846

Query 8890 ATACATGGGACGATGGGACACTTCATCGTCGCACAYTGTCCRCCAGGCGACTACCTCAAG 8949

||||||||||||||||||||||||||||||||||| ||||| ||||||||||||||||||

Sbjct 8847 ATACATGGGACGATGGGACACTTCATCGTCGCACATTGTCCGCCAGGCGACTACCTCAAG 8906

Query 8950 GTTTCGTTCGAGGACGCAGATTCRCACGTGAAGGCATGTAAGGTCCAATACAAGCACGAY 9009

||||||||||||||||||||||| |||||||||||||||||||||||||||||||||||

Sbjct 8907 GTTTCGTTCGAGGACGCAGATTCACACGTGAAGGCATGTAAGGTCCAATACAAGCACGAC 8966

Query 9010 CCATTGCCGGTGGGTAGAGAGAAGTTCGTGGTTAGACCACACTTTGGCGTAGAGCTGCCA 9069

|||||||||||||||||||||||||||||||||||||| |||||||||||||||||||||

Sbjct 8967 CCATTGCCGGTGGGTAGAGAGAAGTTCGTGGTTAGACCCCACTTTGGCGTAGAGCTGCCA 9026

Query 9070 TGCACCTCATACCAGCTGACAACGGCTCCCACCGACGAGGAGATTGACATGCATACACCG 9129

||||||||||||||||||||||| |||||||||||||||||||| |||||||| ||||||

Sbjct 9027 TGCACCTCATACCAGCTGACAACAGCTCCCACCGACGAGGAGATCGACATGCACACACCG 9086

Query 9130 CCAGATATACCGGATCGCACCCTGCTATCACAGACGGCGGGCAACGTCAAAATAACAGCA 9189

||||||||||||||||||||||||||||||||||||||||||||||||||||||||||||

Sbjct 9087 CCAGATATACCGGATCGCACCCTGCTATCACAGACGGCGGGCAACGTCAAAATAACAGCA 9146

Query 9190 GGCGGCAGGACTATCAGGTACAATTGTACCTGYGGCCGTGACAACGTAGGCACTACCAGT 9249

|||||||||||||||||||||||||||||||| |||||||||||||||||||||||||||

Sbjct 9147 GGCGGCAGGACTATCAGGTACAATTGTACCTGTGGCCGTGACAACGTAGGCACTACCAGT 9206

Query 9250 ACTGACAAGACCATCAACACATGCAAGATTGACCAATGCCATGCTGCCGTTACCAGCCAT 9309

||||||||||||||||||||||||||||||||||||||||||||||||||||||||||||

Sbjct 9207 ACTGACAAGACCATCAACACATGCAAGATTGACCAATGCCATGCTGCCGTTACCAGCCAT 9266

Query 9310 GACAAATGGCAATTTACCTCTCCATTTGTTCCCAGGGCTGATCAGACAGCTAGGAGGGGC 9369

||||||||||||||||||||||||||||||||||||||||||||||||||||||||||||

Sbjct 9267 GACAAATGGCAATTTACCTCTCCATTTGTTCCCAGGGCTGATCAGACAGCTAGGAGGGGC 9326

Query 9370 AAAGTGCATGTTCCATTCCCTTTGACTAACGTCACCTGCCGAGTGCCGTTGGCTCGAGCG 9429

||||||||||||||||||||||||||||||||||||||||||||||||||||||||||||

Sbjct 9327 AAAGTGCATGTTCCATTCCCTTTGACTAACGTCACCTGCCGAGTGCCGTTGGCTCGAGCG 9386

Query 9430 CCGGATGTCACCTATGGTAAGAAGGAGGTGACCCTGAGATTACACCCAGATCATCCGACG 9489

||||||||||||||||||||||||||||||||||||||||||||||||||||||||||||

Sbjct 9387 CCGGATGTCACCTATGGTAAGAAGGAGGTGACCCTGAGATTACACCCAGATCATCCGACG 9446

Query 9490 CTCTTCTCCTATAGGAGTTTAGGAGCCGAACCGCACCCGTACGAGGARTGGGTTGACAAG 9549

||||||||||||||||||||||||||||||||||||||||||||||| ||||||||||||

Sbjct 9447 CTCTTCTCCTATAGGAGTTTAGGAGCCGAACCGCACCCGTACGAGGAGTGGGTTGACAAG 9506

Query 9550 TTCTCTGAGCGCATCATCCCAGTGACGGAAGAAGGGATTGAGTACCAGTGGGGCAACAAC 9609

||||||||||||||||||||||||||||||||||||||||||||||||||||||||||||

Sbjct 9507 TTCTCTGAGCGCATCATCCCAGTGACGGAAGAAGGGATTGAGTACCAGTGGGGCAACAAC 9566

Query 9610 CCGCCGGTCCGCCTGTGGGCGCAACTGACGACCGAGGGCAAACCCCATGGCTGGCCACAT 9669

|||||||||||||| |||||||||||||||||||||||||||||||||||||||||||||

Sbjct 9567 CCGCCGGTCCGCCTATGGGCGCAACTGACGACCGAGGGCAAACCCCATGGCTGGCCACAT 9626

Query 9670 GAAATCATTCAGTACTATTATGGACTATACCCCGCCGCCACYATTGCCGCAGTATCCGGG 9729

||||||||||||||||||||||||||||||||||||||||| ||||||||||||||||||

Sbjct 9627 GAAATCATTCAGTACTATTATGGACTATACCCCGCCGCCACCATTGCCGCAGTATCCGGG 9686

Query 9730 GCGAGTCTGATGGCCCTCCTAACTCTAGCGGCCACATGCTGCATGCTGGCCACCGCGAGG 9789

||||||||||||||||||||||||||||||||||||||||||||||||||||||||||||

Sbjct 9687 GCGAGTCTGATGGCCCTCCTAACTCTAGCGGCCACATGCTGCATGCTGGCCACCGCGAGG 9746

Query 9790 AGAAAGTGCCTAACACCATACGCCTTGACGCCAGGAGCGGTGGTACCGTTGACACTGGGG 9849

||||||||||||||||||||||||||||||||||||||||||||||||||||||||||||

Sbjct 9747 AGAAAGTGCCTAACACCATACGCCTTGACGCCAGGAGCGGTGGTACCGTTGACACTGGGG 9806

Query 9850 CTGCTTTGCTGCGCACCGAGGGCGAAYGCAGCATCATTCGCTGAGACTATGGCMTATCTG 9909

|||||||||||||||||||||||||| |||||||||||||||||||||||||| ||||||

Sbjct 9807 CTGCTTTGCTGCGCACCGAGGGCGAACGCAGCATCATTCGCTGAGACTATGGCATATCTG 9866

Query 9910 TGGGACGAGAACAAAACCCTCTTTTGGATGGAATTCGCGGCCCCRGCCGCWGCGCTTGCT 9969

|||||||||||||||||||||||||||||||||||||||||||| ||||| |||||||||

Sbjct 9867 TGGGACGAGAACAAAACCCTCTTTTGGATGGAATTCGCGGCCCCAGCCGCAGCGCTTGCT 9926

Query 9970 TTGCTGGCATGCTGTATCAAAAGCCTGATCTGCTGTTGTAAGCCATTTTCTTTTTTAGTG 10029

||||||||||||||||||||||||||||||||||||||||||||||||||||||||||||

Sbjct 9927 TTGCTGGCATGCTGTATCAAAAGCCTGATCTGCTGTTGTAAGCCATTTTCTTTTTTAGTG 9986

Query 10030 TTACTGAGCCTGGGAGCCTCCGCAAAAGCTTACGAGCACACAGCCACAATTCCGAATGTG 10089

||||||||||||||||||||||||||||||||||||||||||||||||||||||||||||

Sbjct 9987 TTACTGAGCCTGGGAGCCTCCGCAAAAGCTTACGAGCACACAGCCACAATTCCGAATGTG 10046

Query 10090 GTGGGGTTCCCGTATAAGGCTCACATTGAAAGGAATGGYTTCTCGCCCATGACTCTGCAG 10149

|||||||||||||||||||||||||||||||||||||| |||||||||||||||||||||

Sbjct 10047 GTGGGGTTCCCGTATAAGGCTCACATTGAAAGGAATGGCTTCTCGCCCATGACTCTGCAG 10106

Query 10150 CTYGAAGTGGTGGAGACAAGCTTGGAACCCACACTTAACCTGGAGTACATTACCTGCGAA 10209

|| |||||||||||||||||||||||||||||||||||||||||||||||||||||||||

Sbjct 10107 CTTGAAGTGGTGGAGACAAGCTTGGAACCCACACTTAACCTGGAGTACATTACCTGCGAA 10166

Query 10210 TACAAGACGGTGGTCCCTTCGCCATTYATCAAATGTTGCGGAACATCAGAATGCTCATCY 10269

|||||||||||||||||||||||||| ||||||||||||||||||||||||||||||||

Sbjct 10167 TACAAGACGGTGGTCCCTTCGCCATTCATCAAATGTTGCGGAACATCAGAATGCTCATCC 10226

Query 10270 AARGAGCAGCCAGACTACCAATGCAAGGTGTACACGGGYGTATACCCWTTCATGTGGGGT 10329

|| ||||||||||||||||||||||||||||||||||| |||||||| ||||||||||||

Sbjct 10227 AAGGAGCAGCCAGACTACCAATGCAAGGTGTACACGGGTGTATACCCATTCATGTGGGGT 10286

Query 10330 GGAGCCTACTGYTTCTGCGACTCCGAGAACACGCAGCTYAGCGAGGCCTATGTCGACAGG 10389

||||||||||| |||||||||||||||||||||||||| |||||||||||||||||||||

Sbjct 10287 GGAGCCTACTGTTTCTGCGACTCCGAGAACACGCAGCTCAGCGAGGCCTATGTCGACAGG 10346

Query 10390 TCAGACGTTTGCAAACATGATCAYGCATYGGCCTACAAGGCACACACRGCCTCTCTAAAA 10449

||||||||||||||||||||||| |||| |||||||||||||||||| ||||||||||||

Sbjct 10347 TCAGACGTTTGCAAACATGATCACGCATCGGCCTACAAGGCACACACGGCCTCTCTAAAA 10406

Query 10450 GCAACAATCAGGATCAGYTAYGGCACCATCAACCAGACCACCGAGGCCTTCGTYAATGGW 10509

||||||||||||||||| || |||||||||||||||||||||||||||||||| |||||

Sbjct 10407 GCAACAATCAGGATCAGTTATGGCACCATCAACCAGACCACCGAGGCCTTCGTTAATGGT 10466

Query 10510 GAACACGCGGTCAACGTGGGCGGAAGCAAGTTCATCTTTGGACCGATCTCAACAGCTTGG 10569

||||||||||||||||||||||||||||||||||||||||||||||||||||||||||||

Sbjct 10467 GAACACGCGGTCAACGTGGGCGGAAGCAAGTTCATCTTTGGACCGATCTCAACAGCTTGG 10526

Query 10570 TCACCGTTCGACAATAAAATTGTCGTGTATAAAGATGATGTCTACAACCAGGACTTYCCA 10629

|||||||||||||||||||||||||||||||||||||||||||||||||||||||| |||

Sbjct 10527 TCACCGTTCGACAATAAAATTGTCGTGTATAAAGATGATGTCTACAACCAGGACTTCCCA 10586

Query 10630 CCCTACGGATCAGGYCAGCCGGGTAGATTCGGAGACATTCAGAGCAGGACAGTGGAGAGC 10689

|||||||||||||| |||||||||||||||||||||||||||||||||||||||||||||

Sbjct 10587 CCCTACGGATCAGGCCAGCCGGGTAGATTCGGAGACATTCAGAGCAGGACAGTGGAGAGC 10646

Query 10690 AAAGACTTGTATGCCAACACGGCCCTAAAACTCTCAAGACCATCACCCGGGGTTGTGCAT 10749

||||||||||||||||||||||||||||||||||||||||||||||||||||||||||||

Sbjct 10647 AAAGACTTGTATGCCAACACGGCCCTAAAACTCTCAAGACCATCACCCGGGGTTGTGCAT 10706

Query 10750 GTGCCATACACGCAGACACCATCCGGATTTAAATATTGGCTGAAGGAGAAAGGATCTTCA 10809

||||||||||||||||||||||||||||||||||||||||||||||||||||||||||||

Sbjct 10707 GTGCCATACACGCAGACACCATCCGGATTTAAATATTGGCTGAAGGAGAAAGGATCTTCA 10766

Query 10810 TTGAATACAAAGGCCCCTTTTGGCTGCAAGATAAAGACCAATCCAGTCAGAGCCATGGAT 10869

||||||||||||||||||||||||||||||||||||||||||||||||||||||||||||

Sbjct 10767 TTGAATACAAAGGCCCCTTTTGGCTGCAAGATAAAGACCAATCCAGTCAGAGCCATGGAT 10826

Query 10870 TGTGCAGTTGGCAGTATACCTGTGTCGATGGACATACCTGACAGTGCATTCACACGAGTG 10929

||||||||||||||||||||||||||||||||||||||||||||||||||||||||||||

Sbjct 10827 TGTGCAGTTGGCAGTATACCTGTGTCGATGGACATACCTGACAGTGCATTCACACGAGTG 10886

Query 10930 GTAGATGCCCCGGCTGTAACAGACCTGAGCTGCCAGGTAGYGGTCTGTACACACTCCTCC 10989

|||||||||||||||||||||||||||||||||||||||| |||||||||||||||||||

Sbjct 10887 GTAGATGCCCCGGCTGTAACAGACCTGAGCTGCCAGGTAGTGGTCTGTACACACTCCTCC 10946

Query 10990 GATTTCGGAGGAGTTGCCACATTGTCTTACAAAACGGACAAACCCGGCAAGTGCGCTGTC 11049

||||||||||||||||||||||||||||||||||||||||||||||||||||||||||||

Sbjct 10947 GATTTCGGAGGAGTTGCCACATTGTCTTACAAAACGGACAAACCCGGCAAGTGCGCTGTC 11006

Query 11050 CACTCACATTCCAACGTCGCAACGTTGCAAGAGGCGACGGTGGATGTCAAGGAGGATGGC 11109

||||||||||||||||||||||||||||||||||||||||||||||||||||||||||||

Sbjct 11007 CACTCACATTCCAACGTCGCAACGTTGCAAGAGGCGACGGTGGATGTCAAGGAGGATGGC 11066

Query 11110 AAGGTCACAGTGCACTTTTCCACGGCGTCCGCCTCCCCGGCCTTCAAAGTGTCCGTCTGT 11169

||||||||||||||||||||||||||||||||||||||||||||||||||||||||||||

Sbjct 11067 AAGGTCACAGTGCACTTTTCCACGGCGTCCGCCTCCCCGGCCTTCAAAGTGTCCGTCTGT 11126

Query 11170 GACGCAAAAACAACGTGCACGGCGGCGTGCGAGCCTCCAAAAGACCACATCGTCCCTTAT 11229

||||||||||||||||||||||||||||||||||||||||||||||||||||||||||||

Sbjct 11127 GACGCAAAAACAACGTGCACGGCGGCGTGCGAGCCTCCAAAAGACCACATCGTCCCTTAT 11186

Query 11230 GGGGCGAGCCATAACAACCAGGTCTTTCCGGACATGTCAGGAACTGCGATGACGTGGGTG 11289

||||||||||||||||||||||||||||||||||||||||||||||||||||||||||||

Sbjct 11187 GGGGCGAGCCATAACAACCAGGTCTTTCCGGACATGTCAGGAACTGCGATGACGTGGGTG 11246

Query 11290 CAGAGGCTGGCCAGTGGGTTAGGTGGGCTGGCTCTCATCGCGGTGGTTGTGCTGGTCTTG 11349

||||||||||||||||||||||||||||||||||||||||||||||||||||||||||||

Sbjct 11247 CAGAGGCTGGCCAGTGGGTTAGGTGGGCTGGCTCTCATCGCGGTGGTTGTGCTGGTCTTG 11306

Query 11350 GTAACCTGCATAACAATGCGTCGGTAAGCTTTAGTTCAAAGGGCCATATAWACCCCTGAA 11409

|||||||||||||||||||||||||||||||||||||||||||||||||| |||||||||

Sbjct 11307 GTAACCTGCATAACAATGCGTCGGTAAGCTTTAGTTCAAAGGGCCATATAAACCCCTGAA 11366

Query 11410 TAGTAACAAAATATAAAAATTACAAAATATGTAGTTCAAAGGGCTAYACTACCCCTGATT 11469

|||||||||||||||||||||||||||||||||||||||||||||| |||||||||||||

Sbjct 11367 TAGTAACAAAATATAAAAATTACAAAATATGTAGTTCAAAGGGCTATACTACCCCTGATT 11426

Query 11470 AGTAACAAAATAGaaaayyacaaaaaTATGTAGTTAAGTATTATAAGATGTGTAGTTCAA 11529

||||||||||||||||| || ||||||||||||||||||||||||||||||||||||||

Sbjct 11427 AGTAACAAAATAGAAAACCAC-AAAATATGTAGTTAAGTATTATAAGATGTGTAGTTCAA 11485

Query 11530 AGGGCTATAWYACCCCTGATTAGTAACaaaatataaaawtaaaaayaTGTAGTTAAGTAC 11589

||||||||| ||||||||||||||||||||||||||| ||||| ||||||||||||||

Sbjct 11486 AGGGCTATATCACCCCTGATTAGTAACAAAATATAAAAACAAAAATATGTAGTTAAGTAC 11545

Query 11590 TAACCAAYAAGTAGACAAATAKATGCTAACCATATATATAACCAGCTAYAGTATACTATA 11649

||||||| ||||||||||||| |||||||||||||||||||||||||| |||||||||||

Sbjct 11546 TAACCAACAAGTAGACAAATAGATGCTAACCATATATATAACCAGCTATAGTATACTATA 11605

Query 11650 TTTAGYTAAGCAGTTGCAGTAGTTWARAATGTAGTTCAAAGGGCTATACAACCCCTGAAT 11709

||||| ||||||||||||||||| | |||||||||||||||||||||||||||||||||

Sbjct 11606 TTTAGCTAAGCAGTTGCAGTAGTA-AGAATGTAGTTCAAAGGGCTATACAACCCCTGAAT 11664

Query 11710 AGTAAcaaaatacaaaaatactaataaaaattttaaaaatcacaagaaatttaattatca 11769

|||||||||||||||||||||||||||||| |||||||||||| |||||| ||| || |

Sbjct 11665 AGTAACAAAATACAAAAATACTAATAAAAA-TTTAAAAATCACTAGAAATCCAATCATTA 11723

Query 11770 aattattaattGGCTAGCCGAACTCTAAGGAGATGTAGGCGTCCGAACTCTACGGAGATG 11829

||||||||||||||||||||||||||||||||||||||||||||||||||| ||||||||

Sbjct 11724 AATTATTAATTGGCTAGCCGAACTCTAAGGAGATGTAGGCGTCCGAACTCTGCGGAGATG 11783

Query 11830 TAGGACTAAATTCTGCCGAACCCCATAACACCGGGGACGTAGGCGTCTAATTTGtttttt 11889

||||||||||||||||||||||||||||||||||||||||||||||||||||||||||||

Sbjct 11784 TAGGACTAAATTCTGCCGAACCCCATAACACCGGGGACGTAGGCGTCTAATTTGTTTTTT 11843

Query 11890 tAATATTTTAC 11900

|||||||||||

Sbjct 11844 TAATATTTTAC 11854

**Figure S4**. RRV read alignments for SRR8291079**.** (**A**) BWA-mem raw reads from the Fastq file from SRR8291079 aligned to the RRV reference, viewed using the Integrated Genome Viewer; (**B**) Approximately, 0.01% of reads from SRR8291079 aligned to RRV. (**C**) Consensus sequence for RRV reads from SRR8291079 shows 97.63% sequence identity with RRV T48, the prototype mouse adapted, mosquito-derived strain of RRV (Nguyen et al., 2020).


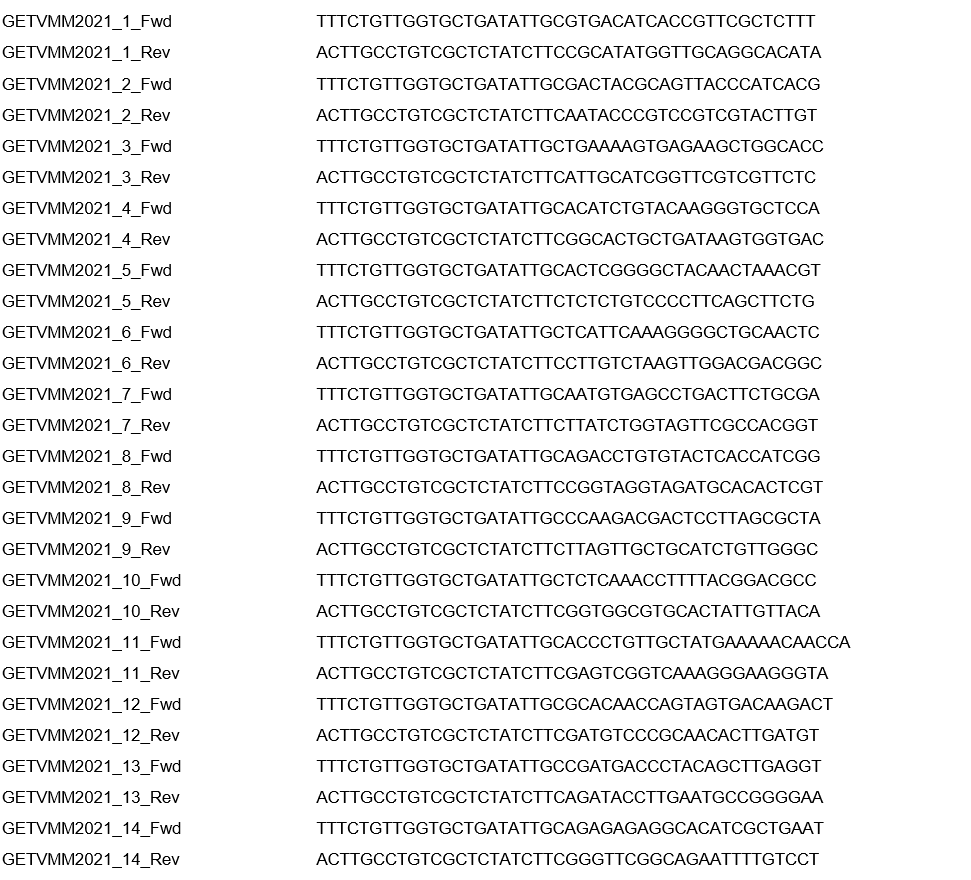


**Fig. S5.** Nanopore sequencing primers.
